# *Nrf2* regulates iron-dependent hippocampal synapses and functional connectivity damage in depression

**DOI:** 10.1186/s12974-023-02875-x

**Published:** 2023-09-21

**Authors:** Ting Zeng, Junjie Li, Lingpeng Xie, Zhaoyang Dong, Qing Chen, Sha Huang, Shuwen Xie, Yuqi Lai, Jun Li, Weixin Yan, YuHua Wang, Zeping Xie, Changlei Hu, Jiayi Zhang, Shanshan Kuang, Yuhong Song, Lei Gao, Zhiping Lv

**Affiliations:** 1 School of Traditional Chinese Medicine, Southern Medical University (SMU), Sha Tai Nan Road No. 1063, Guangzhou, 510515 Guangdong China; 2 Department of Brain Diseases, The Affiliated TCM Hospital of Guangzhou Medical University, Guangzhou, Guangdong China; 3 Department of Hepatology, Cancer Center, Integrated Hospital of Traditional Chinese Medicine, Southern Medical University, Guangzhou, 510315 China; 4 School of Nursing, Guangzhou University of Chinese Medicine, Guangzhou, Guangdong China; 5 The First Affiliated Hospital of Guangzhou University of Chinese Medicine, Guangzhou, Guangdong China; 6 Department of Traditional Chinese Medicine, Guangzhou First People’s Hospital, School of Medicine, South China University of Technology, Guangzhou, 510180 Guangdong China

**Keywords:** Depression, Iron metabolism, *Nrf2*, Synapse damage, Rs-fMR

## Abstract

Neuronal iron overload contributes to synaptic damage and neuropsychiatric disorders. However, the molecular mechanisms underlying iron deposition in depression remain largely unexplored. Our study aims to investigate how nuclear factor-erythroid 2 (NF-E2)-related factor 2 (*Nrf2*) ameliorates hippocampal synaptic dysfunction and reduces brain functional connectivity (FC) associated with excessive iron in depression. We treated mice with chronic unpredictable mild stress (CUMS) with the iron chelator deferoxamine mesylate (DFOM) and a high-iron diet (2.5% carbonyl iron) to examine the role of iron overload in synaptic plasticity. The involvement of *Nrf2* in iron metabolism and brain function was assessed using molecular biological techniques and in vivo resting-state functional magnetic resonance imaging (rs-fMRI) through genetic deletion or pharmacologic activation of *Nrf2*. The results demonstrated a significant correlation between elevated serum iron levels and impaired hippocampal functional connectivity (FC), which contributed to the development of depression-induced CUMS. Iron overload plays a crucial role in CUMS-induced depression and synaptic dysfunction, as evidenced by the therapeutic effects of a high-iron diet and DFOM. The observed iron overload in this study was associated with decreased *Nrf2* levels and increased expression of transferrin receptors (TfR). Notably, inhibition of iron accumulation effectively attenuated CUMS-induced synaptic damage mediated by downregulation of brain-derived neurotrophic factor (BDNF). *Nrf2*^*−/−*^ mice exhibited compromised FC within the limbic system and the basal ganglia, particularly in the hippocampus, and inhibition of iron accumulation effectively attenuated CUMS-induced synaptic damage mediated by downregulation of brain-derived neurotrophic factor (BDNF). Activation of *Nrf2* restored iron homeostasis and reversed vulnerability to depression. Mechanistically, we further identified that *Nrf2* deletion promoted iron overload via upregulation of TfR and downregulation of ferritin light chain (FtL), leading to BDNF-mediated synapse damage in the hippocampus. Therefore, our findings unveil a novel role for *Nrf2* in regulating iron homeostasis while providing mechanistic insights into poststress susceptibility to depression. Targeting *Nrf2*-mediated iron metabolism may offer promising strategies for developing more effective antidepressant therapies.

**Supplementary Information:**

The online version contains supplementary material available at 10.1186/s12974-023-02875-x.

## Introduction

Major depressive disorder (MDD) is a significant neuropsychiatric condition that currently ranks as the third leading cause of global disease burden, with projections from the World Health Organization indicating that it will become the leading cause by 2030 [1]. Despite notable progress in the study of antidepressants, there remains a lack of entirely satisfactory treatments for depression [2]. Therefore, further investigations are necessary to gain a comprehensive understanding of the underlying pathophysiologic mechanisms of depression and to facilitate the development of novel therapeutic compounds. Basic neuroscience research has demonstrated that chronic stress and depressive-like behaviours are linked to a decrease in the size of brain regions responsible for regulating mood and cognition, particularly the reduction of neuronal synapses in the hippocampus [3, 4]. There is substantial evidence indicating that stressful life events lead to changes in synaptic plasticity and a decrease in spine density of pyramidal neurons in the hippocampus, as well as reductions in synaptophysin (SYN) and postsynaptic density protein-95 (PSD95), which contribute to the development of depression [5–7].

Iron is a dichotomous element that possesses both indispensable qualities and the potential to be detrimental to neuronal cells. It is widely acknowledged that an excess of iron in specific regions of the body can result in the continuous production of surplus reactive oxygen radicals and highly oxidative free radicals [8]. The accumulation of iron, accompanied by unregulated generation of reactive oxygen species (ROS) through the Fenton reaction, has the potential to significantly enhance excitotoxicity in glutamatergic neurons, thereby influencing synaptic plasticity and neuronal damage [9, 10]. It is noteworthy that an association exists between elevated iron levels and increased depressive symptoms, suggesting the possibility of iron serving as a potential biomarker for comprehending the pathophysiological mechanism underlying depression [11, 12]. Recent investigations into serum proteomics in depressed patients have revealed abnormal expression of ferritin [11, 12], while a chronic stress depression model in mice has demonstrated a close relationship between hippocampal iron deposition and neuronal degeneration and death [14].

Nuclear factor-erythroid 2 (NF-E2)-related factor 2 (*Nrf2*) is a crucial transcription factor that is sensitive to redox reactions. It plays a significant role in regulating cellular antioxidant responses and cytoprotective and anti-inflammatory genes [15]. Two postmortem studies have shown a reduction in *Nrf2* levels in the hippocampus, prefrontal cortex, and parietal cortex, suggesting that *Nrf2* deficiency is implicated in the development of MDD [16, 17]. Notably, Nrf2 knockout mice exhibit a range of depression-like behaviours [18]. The dysfunction of *Nrf2* results in an imbalance in redox status in neurodegenerative and chronic inflammatory conditions, potentially contributing to a depression-like phenotype [19]. Previous studies have demonstrated that the absence of *Nrf2* suppresses ferroportin 1 transcription in macrophages, leading to iron accumulation and an inflammatory response [20, 21]. Additionally, ferritin light chain (FtL) and ferritin heavy chain (FtH), both transcription targets of *Nrf2*, combine to form ferritin, which effectively sequesters labile iron within its core, preventing its involvement in Fenton reactions [22, 23]. Thus, there is a clear consensus regarding the significant involvement of *Nrf2* in regulating cellular iron homeostasis. However, the impact of *Nrf2*-mediated iron metabolism on depression, brain function, and the distinct brain abnormalities observed in *Nrf2* KO (*Nrf2*^−/−^) mice has yet to be thoroughly investigated. Therefore, it is imperative to explore the mechanisms underlying *Nrf2*-mediated iron metabolism in relation to hippocampal structure and function abnormalities associated with depression, as this will provide valuable insights into potential therapeutic targets for depression.

To investigate the role of *Nrf2*-mediated iron metabolism in depression-like behaviour induced by chronic unpredictable mild stress (CUMS), we conducted in vivo experiments utilizing animal rs-fMRI techniques and biochemical experimental methods to assess the involvement of *Nrf2-*mediated iron metabolism in synaptic plasticity and in the hippocampus. Our findings demonstrate that *Nrf2*^−/−^ mice exhibit diminished functional connectivity, particularly within the hippocampus. Additionally, *Nrf2* plays a pivotal role in regulating both the biochemical imbalances of iron metabolites and the abnormalities in hippocampal structure and function. Deficiency in *Nrf2* results in an excessive accumulation of iron, which in turn contributes to the disruption of synaptic plasticity in the hippocampus and the manifestation of depression-like behaviour. Consequently, our findings offer insights into a potential therapeutic approach for depression through the utilization of *Nrf2*-mediated pathways for iron detoxification.

## Methods

### Animal experiment

Under the Guidelines of the National Institutes of Health for the Care and Use of Laboratory Animals, the mice were bred at the Animal Experimental Centre of Southern Medical University in a specific pathogen-free facility with constant environmental conditions of 23–25 °C, 50 ± 5% humidity, and a 12-h light–dark cycle and were given free access to standard water and food. Six-week-old male *Nrf2*^−/−^ mice (B6.129X1-Nfe212^tm1Ywk/J^, Jackson Laboratory, 017009, Bar Harbour, ME, USA) and wild-type (WT) littermates (18–22 g) were genotyped and allowed to acclimatize to the new environment for 1 week before initiating the experiments. The gene identification results of *Nrf2*^*−/−*^ mice are shown in Additional file 1: Fig. S1A. All animal experiments were approved by the National Institutional Animal Care and Ethical Committee of Southern Medical University (approval number: L2019114).

To establish the association between iron overload and depression, mice were treated with CUMS and iron DFOM (Additional file 1: Fig. S1B). WT mice were randomly separated into 3 groups according to body weight: vehicle, CUMS, and DFOM (100 mg/kg, DFOM, Selleck, S5742). DFOM was dissolved in pyrogen-free normal saline solution at a concentration of 100 mg/mL by intranasal administration three times a week at a dose of 100 mg/kg according to previous studies. In the iron overload experiment (Fig. S1C), WT mice were randomly separated into 4 groups according to body weight: control diet vehicle, control diet CUMS, high-iron diet vehicle and high-iron diet CUMS. At the beginning of CUMS, high-iron diet mice were fed a standard AIN93 diet containing 2.5% carbonyl iron (TP0457G, TROPHIC Animal Feed High-Tech Co. Ltd, China) to establish an iron-overloaded model, while other group mice were fed a control diet (45 ppm of iron, LAD3001G).

To test the effect of *Nrf2* on depression, we used the *Nrf2* activator oltipraz to treat CUMS mice (Additional file 1: Fig. S1D). WT mice were randomly separated into 3 groups according to body weight: Control, CUMS, and oltipraz (10 mg/kg, Selleck, S7864). Vehicle and CUMS mice received an equal volume of vehicle. Oltipraz was dissolved in 2% dimethyl sulfoxide, 40% PEG300 and 5% Tween 80 at a concentration of 1 mg/mL and administered intraperitoneally at a dose of 10 mg/kg once per day. Additionally, WT mice were randomly divided into 2 groups: control and CUMS, while *Nrf2*^−/−^ mice were randomly divided into 2 groups: *Nrf2*^−/−^ control and *Nrf2*^−/−^ CUMS (Additional file 1: Fig. S1E). The CUMS group and *Nrf2*^−/−^ CUMS group were exposed to CUMS for 6 weeks. In the rs-fMRI experiment, after the behavioural experiment, rs-fMRI of anaesthetized mice in each group was performed.

Test animals were fasted overnight from 8 p.m. to 8 a.m. prior to euthanasia and dissection and then euthanized with 1.25% sodium pentobarbital 24 h after behaviour data collection. After anaesthesia, blood was immediately collected by cardiac puncture for biochemical analysis. Some of the mice were perfused and the hippocampus was fixed for paraffin and frozen sectioning, and the hippocampus of other mice was quickly frozen with liquid nitrogen for further study.

### CUMS procedure

Mice were exposed to a diverse range of unpredictable mild stressors for 6 weeks. The social stressors listed below were randomly administered once or twice daily over the course of a week and were repeated across the 6-week experiment. The control mice were left undisturbed in their home cages with the exception of general handling, vehicle treatment and behavioural tests. The weight of the mice was measured twice a week, and the average value was taken.

(1) 24 h of food deprivation; (2) 24 h of water deprivation; (3) 24 h of no bedding; (4) 12 h of 45° cage tilt; (5) a reversed light–dark circadian cycle (12:12 h); (6) 24 h of wet bedding (~ 200 ml of clean water in 100 g of sawdust bedding, which was enough to not cause pooling of water); (7) 4 h of physical restraint; (8) 30 min of shaking; and (9) flash exposure overnight.

### Behavioural tests

The behavioural tests were conducted and recorded by 2 well-trained and experienced observers who were blind to the animal experiments. At the end of the 6th week, all animals were subjected to behavioural tests. The sucrose preference test (SPT) was carried out 3 times: before the experiment (baseline), in the middle of the experiment (3rd week), and at the end of the experiment (6th week). The tail suspension test (TST), forced swim test (FST) and open field test (OFT) were performed after the final SPT.

*Sucrose preference test (SPT)* The SPT was used to evaluate CUMS-induced anhedonia. Before the test, the mice were trained to adapt to two bottles of water to drink for 48 h, of which one bottle was filled with 1% sucrose solution (w/v) and one bottle was filled with pure water in each cage. After acclimatization, the mice were deprived of food and water for 24 h and then exposed to a free choice between two preweighed plastic bottles containing water or 1% sucrose solution for 24 h. The consumption of sucrose and water was calculated by measuring the weight change of the liquid. The sucrose preference was calculated as sucrose consumption/(sucrose consumption + water consumption) × 100%.

*Tail suspension test (TST)* The TST was used to analyse the depressive behaviour of mice. The mice were separated from each other and then suspended with a medical adhesive cloth approximately 1 cm away from the tail tip of the mice. The mice were observed for a period of 6 min and the total immobile time in the last 4 min was calculated. The mice were defined as immobile only when they gave up any struggle and remained still.

*Forced swim test (FST)* Another test, the FST, was performed to analyse despair-like behaviour of mice. In the test, the mice were forced to swim in an open glass cylinder (14 cm inner diameter, with water level 25 cm deep) filled with water at 23 ± 1 °C. The mice were gently put into the water, and swimming behaviour was recorded for 6 min. The total immobile time in the last 4 min was scored. Immobility was defined as stopping swimming and staying motionless, except for doing slight necessary movements to keep the head afloat.

*Open-field test (OFT)* The voluntary movement and behaviours of the mice were evaluated using the OFT. Mice were gently placed at the centre of an open field apparatus consisting of a 40 cm × 40 cm × 40 cm grey square box divided into 4 quadrants and observed for 6 min. The apparatus was cleaned with 75% ethanol and dried before each mouse was tested. The time in the centre (an area of 20 cm × 20 cm in the centre of the box) was also traced and recorded by Smart 3.0 (Panlab Spain).

### s-fMRI data acquisition and analysis for mice

rs-fMRI data acquisition is described in Additional file 1. A 7.0 T small animal MRI scanner (70/16PharmaScan, Bruker Biospin GmbH, Germany) equipped with a receive-only mouse brain surface coil suitable for the mice was used to perform rs-fMRI scanning. For the fMRI handling procedures, adult male mice were anaesthetized with 3.5% isoflurane for 5 min and endotracheally intubated. Then, the mice were mechanically ventilated with isoflurane 0% ~ 0.3%. The muscle relaxants pancuronium bromide (0.2 mg/kg I.P.) and dexmedetomidine (0.04 mg/kg/h S.C.) were applied at the same time. During the examination, the mice were placed on a plastic cradle with the head fixed with a tooth bar and plastic screws in the ear canals. The physiological status of all animals was monitored throughout the imaging procedure. The ventilator (Kent Scientific) precisely monitored the breathing rate of the mice, and the SAII heater system monitored the normal body temperature of the mice. Moreover, the blood oxygen saturation and heart rate level were monitored in real time, making the animal breathing rate = 80/min; the rectal temperature was 37 ± 0.1 °C; the heart rhythm was 260 ~ 360; and oxygen saturation was > 95%. Data acquisition was started with a T2 TurboRARE sequence (repetition time/echo time = 2500/35 ms, matrix size = 256 × 256, field of view = 16 × 16 cm^2^, slice thickness = 0.5 mm, slices = 22). After the anatomical image was acquired, an echo plane imaging (EPI) sequence was used for the fMRI scan with the following parameters: repetition time/echo time = 750/15 ms, matrix size = 64 × 64, field of view = 16 × 16 cm^2^, slice thickness = 0.5 mm, slices = 22, repetitio*n* = 480, and total scanning time = 6 min.

For the preanalysis of rs-fMRI, the following processing was performed. (1) The scalp signal was manually removed using ITK-SNAP software. (2) Slice timing correction: the analysis time of each brain layer of each subject was corrected. (3) Head movement correction: six parameters were used to eliminate tiny head movements. (4) Spatial standardization: the brain images of all tested mice were standardized to templates to eliminate the differences between subjects. The images were linearly registered to approximate the brain space of C57Bl6, which is a previously established template specific for the brain anatomy of C57 mice [24]. See Additional file 1 for detailed procedures of data processing. (5) Gaussian smoothing: Gaussian smoothing was used to standardize spatially normalized data, and full width at half maximum (FWHM) was used to further eliminate noise, which made the statistical data analysis have a normal distribution. (6) Nonlinear drift: the image of linear time-varying information was eliminated to avoid errors caused by the equipment. (7) The frequency of temporal bandpass filtering was 0.0007–0.1 Hz. (8) The FC value and other indicators of the seed region of interest (ROI) in each group of brains were further analysed and calculated by MATLAB (MATLAB R2014a, The MathWorks Inc. Natick, MA, USA).

FC analysis was conducted using seed-based analysis and ROI analysis.

*Seed-based analysis* Two 4 × 4 voxel regions were defined as the ipsilateral and contralateral seeds. Twelve pairs of seeds were selected, including the hippocampus CA1 (CA1), hippocampus CA3 (CA3), dentate gyrus (DG), PreSubiculum (PreSub), subiculum (Sub), ventral dentate gyrus (vDG), basolateral amygdala (BLA), infralimbic area (IL), striatum (Str), central amygdalar nucleus (CeA), pretectal area (Pre), and pallidum (PAL). The respective reference time course is the regionally averaged time course of the voxel within each seed. The rs-fMRI connectivity maps for each seed were generated by the Pearson correlation coefficient (CC) calculation between the reference time course and the time course of every other voxel. We quantified the interhemispheric FC for each region by averaging the CC value of the corresponding ipsilateral and contralateral seed and then performed Fisher z score transformation, while we quantified the intrahemispheric FC by averaging the CC value of the seed itself.

*ROI analysis* Forty-six regions of interest (ROIs) were manually defined: the bilateral CA1, CA3, DG, PreSub, Sub, vDG, CeA, orbital area (ORB), piriform area (PL), infralimbic area (IL), anterior cingulate area (ACA), retrosplenial area (RSP), caudoputamen (CPu), nucleus accumbens (NAc), pallidum (PAL), lateral septal nucleus (LSN), medial habenula (MHB), lateral habenula (LHB), ventral medial nucleus of the thalamus (vTHA), lateral posterior nucleus of the thalamus (dTHA), substantia nigra (SN), pontine reticular nucleus (PRN) and pedunculopontine nucleus (PPN). BOLD time series were first extracted from the 46 ROIs, and then Pearson correlation analysis in pairs was performed to determine the temporal correlation between the extracted signal and the time series from all other brain voxels, generating the functional correlation maps. The correlations were transformed by Fisher z score transformation for group statistics. To assess statistically significant differences between the two groups, we performed a two-sample t test and then corrected by the false discovery rate (FDR) for multiple comparisons.

### Western blot analysis of the hippocampus

Frozen hippocampal tissues were homogenized in ice-cold RIPA buffer (Sigma, USA) containing 1% protein inhibitor (Sigma, USA) and 1% phosphatase inhibitor (Sigma, USA). After centrifugation at 12,000 rpm at 4 °C for 15 min, the supernatant was collected for quantification by a BCA protein assay kit (Thermo Fisher Scientific) and then denatured in loading buffer. Equal amounts of protein (50 µg) were separated on 10% gels by SDS‒PAGE and transferred to polyvinylidene fluoride (PVDF) membranes (Millipore, Billerica, MA, USA). The membrane was placed in 5% foetal bovine serum albumin (BSA) in washing buffer (Tris-buffered saline containing 0.1% v/v Tween-20), blocked at room temperature for 2 h, and then incubated overnight at 4 °C with primary antibodies [GAPDH (rabbit, 1:1000, Cell Signaling Technology, USA), β-actin (rabbit, 1:1000, Santa Cruz, USA), BDNF (1:1000, Abcam, UK), Nrf2 (rabbit, 1:1000, Proteintech, USA), DMT1 (rabbit, 1:1000, Abcam, UK), TfR (rabbit, 1:1000, Abcam, UK), Tf (rabbit, 1:1000, Abcam, UK), PSD95 (mouse, 1:200, Abcam, UK), and SNAP25 (rabbit, 1:1000, Abcam, UK)] followed by incubation with horseradish peroxidase-linked secondary antibodies for 2 h at 4 °C. Chemiluminescence detection was measured using ECL reagents (Millipore, Billerica, MA, USA). The bands were scanned, and their densities were analysed by ImageJ.

### Immunohistochemistry (IHC) of the hippocampus

Paraffin-embedded brain tissue sections were deparaffinized in dimethylbenzene and rehydrated in ethanol. The activity of endogenous peroxidase was inactivated by incubating in 3% H_2_O_2_ at 37 °C for 10 min. The sections were immersed in citrate buffer (pH = 6.0), heated to 100 °C and cooled at room temperature for antigen recovery. After nonspecific binding sites were blocked in 5% goat serum containing Triton X-100 (0.2% v/v) for 2 h, the sections were then combined with GluR1 (1:200, Santa Cruz, USA), Iba1 (1:1000, Abcam, UK), and SYN (1:200, Abcam, UK) antibodies in IHC antibody diluent overnight at 4 °C. After washing in phosphate-buffered saline (PBS), horseradish peroxidase-conjugated secondary anti-rabbit antibodies (Gene Tech, USA) were used for 2 h. The reaction was developed with a 3,3′-diaminobenzidine (DAB, Gene Tech, USA) kit and counterstained with haematoxylin. Finally, images were taken on a microscope (Olympus, Tokyo, Japan) at a magnification of 200 ×, and the averaged optical densities (AOD) of the target protein as well as the number of Iba1-labelled microglia were calculated with ImageJ. The data were analysed by GraphPad Prism 5.0.

### Immunofluorescence of the hippocampus

The process was similar to IHC. Brain samples were quickly obtained and fixed in a 4% PFA solution. Then, 10-μm-thick hippocampal coronal sections were attached to glass slides, dried for 1 h at RT and stored at − 20 °C until use. Sections were permeabilized with prechilled methanol at -20 °C for 10 min and then blocked with 5% normal goat serum solution (Sigma, USA) at room temperature for 1 h. Subsequently, the sections were incubated overnight with anti-BDNF (1:200, Abcam, UK), Nrf2 (1:100, Proteintech, USA), PSD95 (1:100, Santa Cruz, USA), FtL (1:100, Proteintech, USA), TfR (rabbit, 1:100, Abcam, UK), Tf (rabbit, 1:100, Abcam, UK), NeuN (1:100, Abcam, UK) and Iba1 (1:100, Abcam, UK) primary antibodies at 4 °C. The next day, the sections were incubated with Alexa Fluor 488- or 594-conjugated anti-rabbit or anti-mouse IgG secondary antibodies (1:400, Invitrogen). The cell nuclei were stained with 4ʹ,6-diamidino-2-phenylindole (DAPI, Solarbio Life Science, China) for 10 min and then cover-slipped with anti-fluorescence quencher (Dako Denmark). A fluorescence microscope (C2 +, Nikon, Japan) or laser scanning confocal microscope was used to visualize immunofluorescence (IF) images of the slices. ImageJ software (National Institutes of Health, USA) was used to analyse the images.

### Mouse serum iron assay

The whole blood samples were placed at room temperature for 2 h or 4 °C overnight and then centrifuged at 3000 rpm at 4 °C for 15 min. The supernatant was stored at -80 °C. After the serum was thawed, the corresponding working reagents and the parameters on the automatic biochemical analyser (Radu, Shenzhen, China) were prepared according to the instructions, and then the sample was loaded and automatically measured using the automatic biochemical analyser.

### Golgi staining

Golgi staining was performed using the FD Rapid Golgi Stain kit (FD Neuro Technologies). The brains of the mice were freshly dissected and immersed in solutions A and B at room temperature for 2 weeks and then transferred into solution C for 24 h at 4 °C in the dark. The brains were sliced at a thickness of 150 μm using a vibratome (Leica). Finally, the brain slice was stained with solutions D and E. Images of hippocampal neurons were taken by the All-in-One fluorescence microscopy imaging system BZ-X800E. We selected 15–20 neurons in the CA1 pyramidal cell layer of the hippocampal region of each mouse and analysed spine number on the 2nd or 3rd apical dendrite branch. We only counted the spine number from a single branch in one neuron.

### Mouse hippocampal tissue iron assay

The ferrous (Fe^2+^) iron and total iron concentrations in the hippocampus of mice were assayed by an Iron Assay Kit (#ab83366, Abcam) in accordance with the manufacturer’s instructions.

### Transmission electron microscopy (TEM)

After the mice were anaesthetized, the whole brain was quickly removed and placed in prechilled 2.5% glutaraldehyde to rapidly separate the hippocampus. Then, the hippocampus was cut into approximately 1 mm^3^ pieces. Then, the hippocampus was incubated in 2.5% glutaraldehyde at room temperature for 1 h and then incubated overnight at 4 °C. The samples were washed with PBS, fixed in 1% osmotic acid at 4 °C for 3 h, dehydrated in ethanol and acetone, and embedded in epoxy resin for 4 h. Finally, the prepared tissue was cut into 70 nm sections, stained with uranyl acetate and lead citrate, and then observed by a Hitachi H-7500 TEM.

### Statistical analysis

Statistical analyses were performed with MATLAB for the fMRI experiments and with GraphPad Prism 8.0 software for other experiments in this study. All data contain at least *n* = 3 per group and are presented as the mean ± standard error of the mean (SEM). All experiments were performed at least three times. “At least three times” represents at least three independent individuals in each group. Moreover, at least three independent repeated experiments, such as WB experiments and staining experiments, were performed. Statistical analysis was performed by using an unpaired two-tailed t test to compare mean values or one-way ANOVA to evaluate the statistical significance of differences in multigroup experiments, followed by Tukey’s multiple-comparisons test on dependent experimental designs. Body weight data were analysed by two-way repeated measures analysis of variance. To investigate whether a high-iron diet or *Nrf2* genetic ablation could potentiate the effect of CUMS induction, two-way ANOVA was used to analyse the relevant indicators, followed by Bonferroni’s post hoc test. For all analyses, *P* values < 0.05 were considered statistically significant and annotated as follows: *P < 0.05, **P < 0.01, ***P < 0.001, ****P < 0.0001. The exact sample size for each experimental condition is represented in the figures as symbols.

## Results

### Iron accumulation was associated with hippocampal functional connectivity dysfunction in CUMS mice

**Fig. 1:**
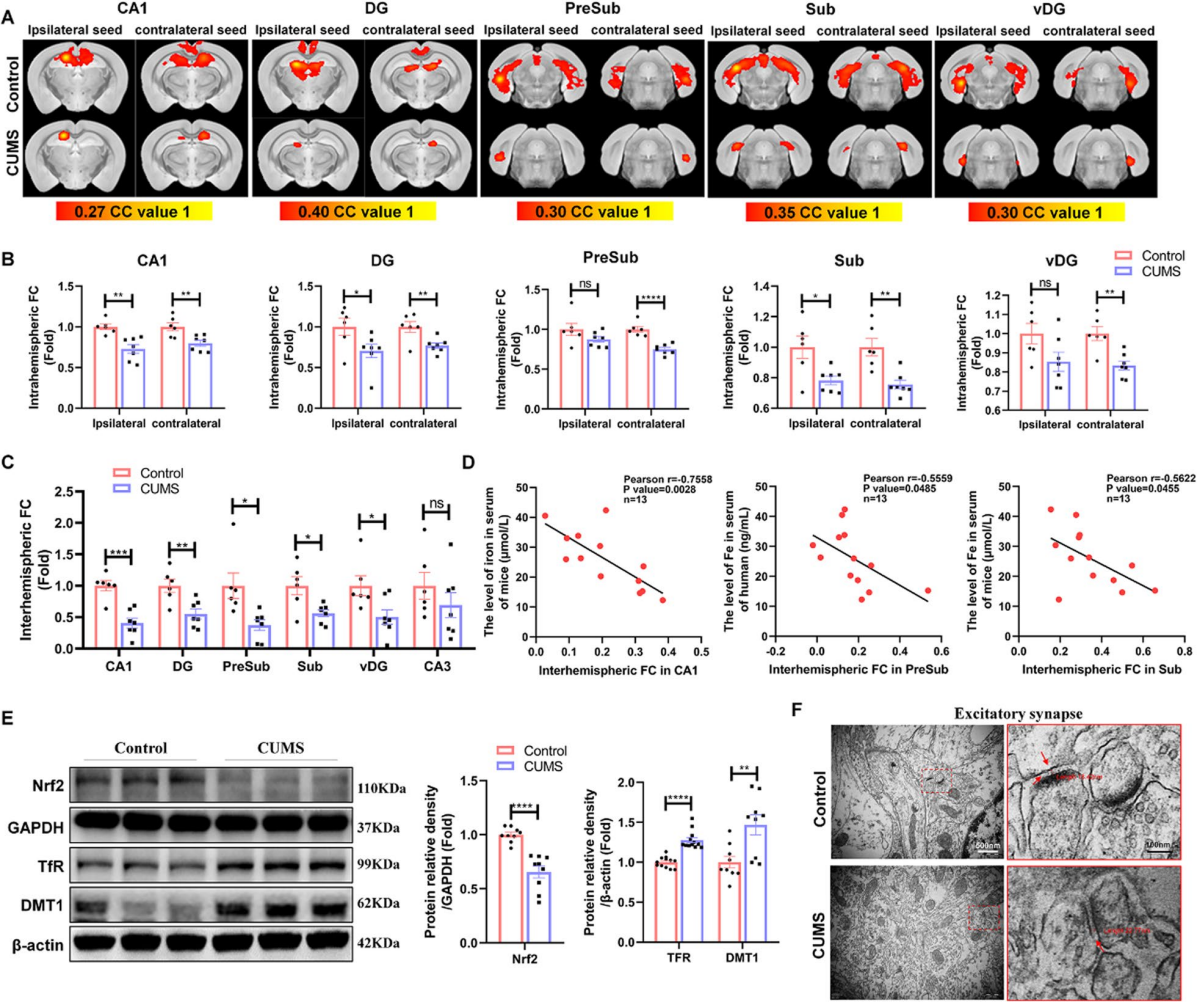
Iron accumulation was associated with hippocampal functional connectivity dysfunction in CUMS mice. **A** Seed-based analysis represented by functional connectivity maps for WT and CUMS mice. The strength of connectivity for the seed region, indicated above each image, is mapped by a colour scale representing the correlation coefficient (CC) value. (Control *n* = 6 and CUMS *n* = 7; abbreviations: CA1 = field of CA1 in the hippocampus, DG = dentate gyrus, PreSub = presubiculum, Sub = subiculum, vDG = ventral dentate gyrus). **B** Average interhemispheric functional connectivity for CA1, DG, PreSub, Sub, and vDG in Control and CUMS mice. **C** Intrahemispheric functional connectivity for the ipsilateral and contralateral seeds of CA1, DG, PreSub, Sub, and vDG in control and CUMS mice. **D** Pearson linear correlation tests for Fe levels in serum and interhemispheric FC in CA1, PreSub, and Sub. **E** Western blot analysis of the relative density ratios of TfR, *Nrf2* and DMT1 expression in the hippocampus of the control and CUMS groups. GAPDH and β-Actin served as a loading control. The density of β-actin, GAPDH, TfR, *Nrf2* and DMT1 protein was measured using ImageJ software. (*Nrf2* and DMT1: *n* = 9/group; TfR: *n* = 12/group). **F** Electron microscopy image of excitatory/asymmetric spines (arrows point to the synaptic cleft, scale bars, 500 nm, *n* = 3 mice/group). Bars represent the mean ± SEM; statistical analysis was performed by using an unpaired two-tailed t test. *P < 0.05, **P < 0.01, ***P < 0.001, and ****P < 0.0001

To investigate the neuronal alterations in the whole brain of mice exposed to CUMS, a seed-based analysis was performed to analyse the functional connectivity of CUMS mice. The results revealed a reduction in both intrahemispheric and interhemispheric functional connectivity in specific regions of the hippocampus, including the CA1, dentate gyrus (DG), presubiculum (PreSub), subiculum (Sub), and ventral dentate gyrus (vDG), in CUMS mice, showing a decrease in the spatial extent of connectivity maps (Fig. 1A–C), which indicated that neuronal function in the hippocampus was altered after CUMS. However, there were no observed alterations in intrahemispheric and interhemispheric functional connectivity in subregions of the amygdala, striatum and prefrontal cortex, except for the weakening of interhemispheric functional connectivity in the basolateral amygdala (BLA) (Additional file 1: Fig. S2A and B). Attractively, Person correlation analysis indicated that the interhemispheric functional connectivity in CA1, PreSub and Sub (Fig. 1D), but not in DG (Additional file 1: Fig. S2C), vDG (Additional file 1: Fig. S2D) and BLA (Additional file 1: Fig. S2E), showed a negative relationship with serum iron levels in mice. This indicates that the elevation of serum iron levels may be closely related to weakened functional connectivity in the hippocampus, especially in the CA1, PreSub and Sub regions. Consequently, we detected the expression of *Nrf2* protein and the iron metabolism-related proteins transferrin receptor (TfR) and divalent metal transporter 1 (DMT1) in the hippocampus. In CUMS mice, an increase in hippocampal TfR and DMT1 was observed, along with a decrease in *Nrf2* protein (Fig. 1E), which meant a significant accumulation of iron in the hippocampus of mice with CUMS-induced depression. Ultrastructural changes in the hippocampus revealed that CUMS blocked the formation and maturation of excitatory synapses, resulting in abnormal structures characterized by a decrease in the number of excitatory synaptic vesicles, a widened synaptic cleft, and a thinned PSD (Fig. 1F). Therefore, we hypothesized that CUMS may induce hippocampal iron overload, which in turn contributes to impairments in functional connectivity and synaptic structures.

### Alleviation of iron overload attenuated depression-like behaviours by ameliorating synaptic damage and activating *Nrf2*

**Fig. 2:**
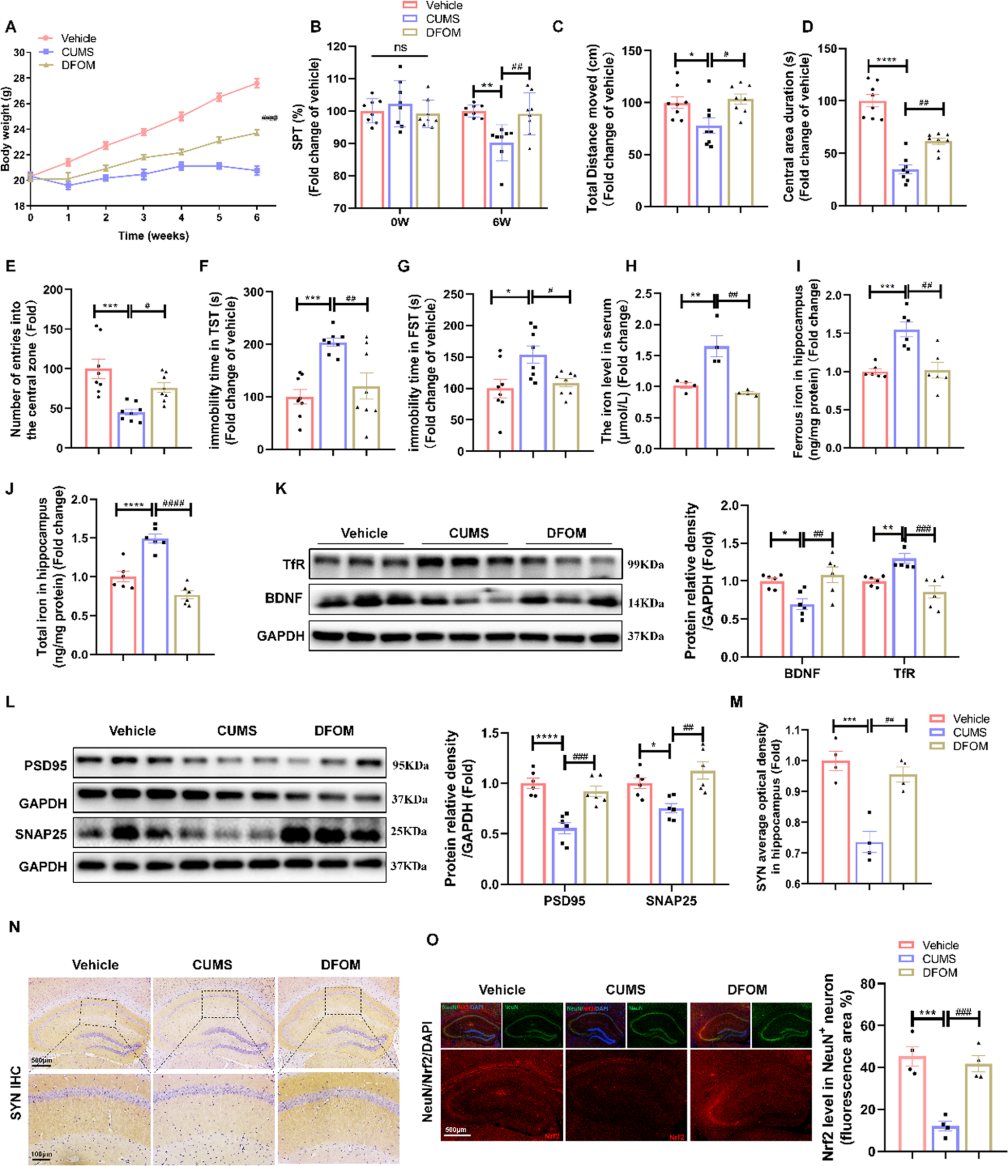
Alleviation of iron overload attenuated depression-like behaviours by ameliorating synaptic damage and activating *Nrf2.* **A** Body weight changes in mice during the CUMS procedure in the vehicle, CUMS and DFOM groups (*n* = 8/group). **B** SPT in each group (*n* = 8/group). **C**–**E** OFT in each group (*n* = 8/group). **F** and **G** TST and FST in each group (all Groups *n* = 8/group).** H** The level of Fe in the serum of mice (*n* = 4/group). **I** and** J** Ferrous iron and total iron in the hippocampus of the different indicated groups (*n* = 6/group). **K** Western blot analysis of the relative density ratios of TfR and BDNF expression in the hippocampus of each group (*n* = 6/group). GAPDH served as a loading control. The density of GAPDH, TfR and BDNF protein was measured using ImageJ software. **L** Western blot analysis of the relative density ratios of PSD95 and SNAP25 expression in the hippocampus of each group (*n* = 6/group). GAPDH served as a loading control. The density of GAPDH, PSD95 and SNAP25 protein was measured using ImageJ software.** M** and** N** Immunohistochemical staining of SYN (sepia) in the hippocampus (*n* = 4/group; scale bars, 100 μm).** O** Immunofluorescence of the hippocampus costained with *Nrf2* (red) and the mature neuron marker NeuN (green) (*n* = 4/group; scale bars, 500 μm). All images of immunostaining were analysed with ImageJ software. Bars represent the mean ± SEM. Bars represent the mean ± SEM. Statistical analysis was performed by one-way ANOVA with Tukey’s post hoc test. Body weight data were analysed by two-way repeated measures analysis of variance. * Represents comparison with the control group; # represents comparison with the CUMS group. *P < 0.05, **P < 0.01, ***P < 0.001, and ****P < 0.0001

To explore the pathological role of iron overload in depression in vivo, we tested the effect of the administration of the iron chelator DFOM. Behavioural results showed that DFOM attenuated the depression-like behaviours in mice induced by CUMS, as measured by the sucrose preference ratio (Fig. 2A) and body weight (Fig. 2B). Additionally, in the open field test, mice in the DFOM group had an increased CUMS-induced reduction in total distance moved (Fig. 2C), time spent in the central arena (Fig. 2D), and number of entries into the central zone (Fig. 2E). DFOM attenuated the CUMS-induced increase in immobility time in the TST (Fig. 2F) and FST (Fig. 2G). Additionally, CUMS resulted in elevated serum iron levels (Fig. 2H), as well as increased ferrous iron (Fig. 2I) and total iron (Fig. 2J) in the hippocampus. However, these iron abnormalities were ameliorated following treatment with DFOM, indicating a potential alleviation of iron overload in the hippocampus. In addition, we found that DFOM significantly reversed the reduction in TfR expression associated with iron overload (Fig. 2K). The role of brain-derived neurotrophic factor (BDNF) in synaptic structure and function is widely recognized. DFOM treatment significantly activated the downregulation of BDNF levels in the hippocampus of CUMS mice (Fig. 2K). In addition, WB and immunostaining revealed significant reductions in PSD95, SNAP25 and SYN expression in the hippocampus following CUMS treatment, whereas an increase in expression was observed in the DFOM-treated group (Fig. 2L–N). These results confirmed that the stabilization of iron homeostasis ameliorated hippocampal synaptic dysfunction by elevating BDNF levels in CUMS mice. Remarkably, immunofluorescence colocalization staining showed that CUMS caused a decrease in *Nrf2* expression in hippocampal cells labelled with neuron-specific nuclear protein (NeuN), which was significantly upregulated after DFOM treatment (Fig. 2O). Consequently, we speculated that the upregulation of Nrf2 induced by DFOM may play a crucial role in preserving neuronal iron homeostasis.

### Iron overload contributed to depression-like behaviour and synaptic plasticity impairment in mice

**Fig. 3:**
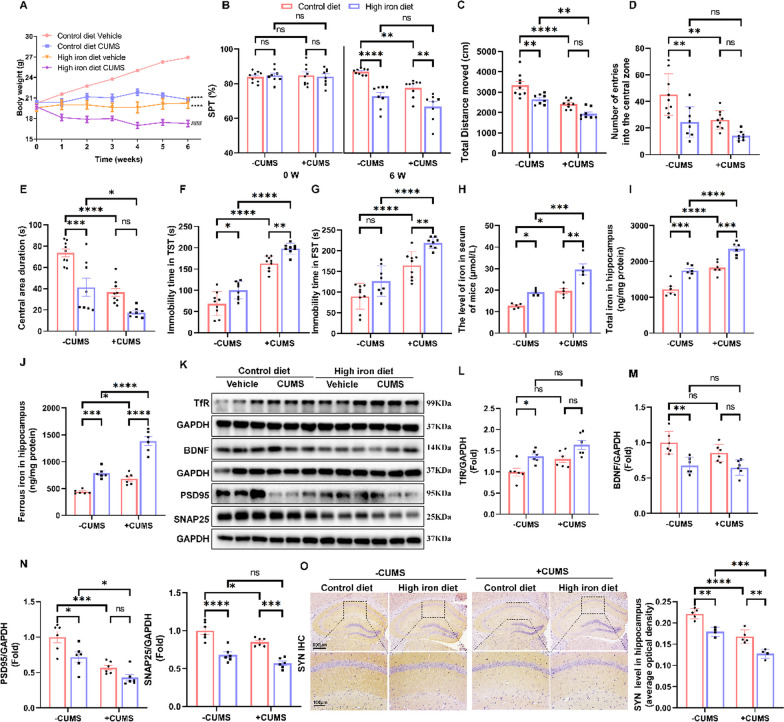
Iron overload contributed to depression-like behaviour and synaptic plasticity impairment in mice. **A** Body weight changes in mice during the CUMS procedure in the control diet vehicle, control diet CUMS, high-iron diet vehicle and high-iron diet CUMS groups (*n* = 8–9/group). **B** SPT in each group (*n* = 8–9/group). **C**–**E** OFT in each group (*n* = 8–9/group). **F** and **G** TST and FST in each group (all Groups *n* = 8–9/group).** H** The level of Fe in the serum of mice (*n* = 5/group). **I** and** J** Ferrous iron and total iron in the hippocampus of the different indicated groups (*n* = 6/group).** K–L** Western blot analysis of the relative density ratios of TfR, BDNF, PSD95 and SNAP25 expression in the hippocampus in each group. GAPDH served as a loading control. The density of each protein was measured using ImageJ software (*n* = 6/group). **O** Immunohistochemical staining of SYN (sepia) in the hippocampus (*n* = 5/group; scale bars, 100 μm). All images of immunostaining were analysed with ImageJ software. Bars represent the mean ± SEM. Statistical analysis was performed by two-way ANOVA with Bonferroni’s post hoc test. * Represents comparison with the control (vehicle) group, # represents comparison with the model group. *P < 0.05, **P < 0.01, ***P < 0.001, and ****P < 0.0001

To further explore the pathological role of iron overload in depression, mice were fed a high-iron diet to establish an iron overload model. Behavioural results showed that the high-iron diet significantly reduced body weight as well as sucrose preferences in both control and CUMS mice (Fig. 3A and B). The open field test also showed that the high-iron diet reduced the total distance moved, the number of entries into the central area, and central area duration for both control and CUMS mice (Fig. 3C–E). Additionally, the high-iron diet also increased the immobility time for both control and CUMS mice in the TST and FST (Fig. 3F and G). Overall, CUMS mice exhibited depressive-like behaviour compared to the control group, whereas CUMS mice in the high-iron state exhibited more severe depressive-like behaviour compared to the high-iron diet controls. There was no significant interaction observed between CUMS and the high-iron diet. Additionally, the CUMS group exhibited increased levels of iron in serum (Fig. 3H), hippocampal total iron (Fig. 3I), ferrous iron (Fig. 3J) and TfR protein (Fig. 3K and L) when compared to the control mice. Similarly, the high-iron diet CUMS group showed increased levels of these factors compared to the high-iron diet controls. The high-iron diet aggravated iron deposition in both CUMS and control mice. To verify the role of iron overload in hippocampal synaptic plasticity, immunostaining and Western blot analysis were performed to visualize proteins of interest. As expected, the results from both western blotting and immunostaining demonstrated a significant decrease in BDNF (Fig. 3K and M), PSD95, SNAP25 (Fig. 3K and N) and SYN (Fig. 3O) in mice fed a high-iron diet, regardless of CUMS treatment. Furthermore, astrocytes were activated after treatment with CUMS and a high-iron diet, accompanied by upregulation of TfR and Tf in astrocytes, indicating enhanced iron absorption by astrocytes in the case of neuronal iron overload (Fig. S6). The above results suggested that iron deposition in the hippocampus has a significant detrimental effect on synapses, resulting in depressive behaviour in mice.

### Induction of *Nrf2* by oltipraz prevented CUMS-induced depression-like behaviours and iron deposition by TfR inhibition

**Fig. 4:**
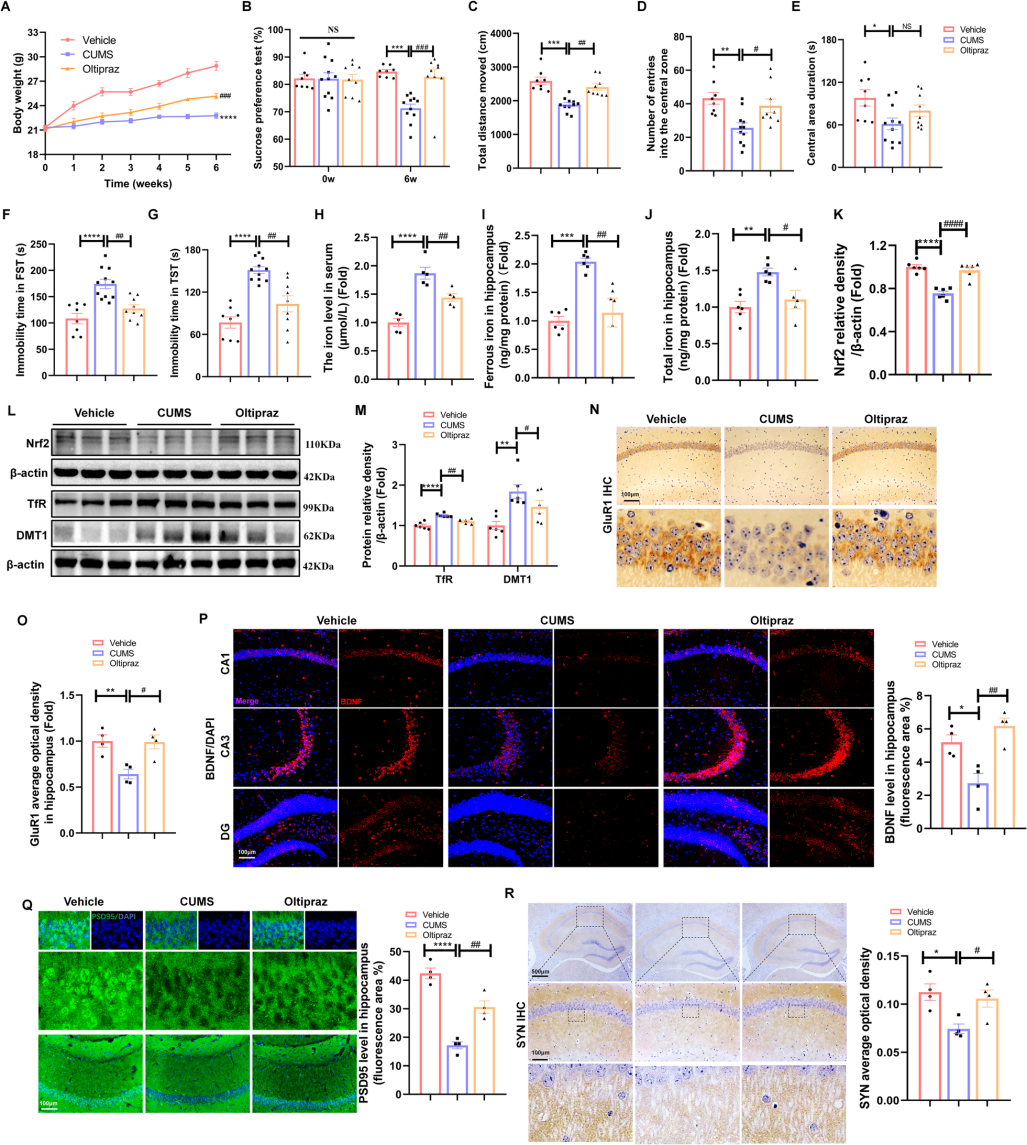
Induction of *Nrf2* by Oltipraz prevented CUMS-induced depression-like behaviours and iron deposition by TfR inhibition. **A**–**G** Body weight, SPT, OFT, TST and FST in the different indicated groups (Control *n* = 8, CUMS *n* = 11, Oltipraz *n* = 9). **H** The level of Fe in the serum of mice (Control *n* = 10, CUMS *n* = 9, Oltipraz *n* = 7). **I** and **J** Ferrous iron and total iron in the hippocampus of the different indicated groups (*n* = 5/group). **K**–**M** Western blot analysis of the relative density ratios of *Nrf2*, TfR and DMT1 expression in the hippocampus. β-Actin served as a loading control. The density of β-actin, *Nrf2*, TfR and DMT1 protein was measured using ImageJ software. (*n* = 6/group). **N** and **O** Immunohistochemical staining of GluR1 (sepia) in the hippocampus (*n* = 4/group; scale bars, 100 μm). **P** Immunofluorescence staining of BDNF in the hippocampus (*n* = 4/group; scale bars, 100 μm). **Q** Immunofluorescence staining of the astrocyte marker PSD95 in the hippocampus (*n* = 4/group; scale bars, 100 μm). **R** Immunohistochemical staining of SYN (sepia) in the hippocampus (*n* = 4/group; scale bars, 100 μm). All images of immunostaining were analysed with ImageJ software. Bars represent the mean ± SEM. Statistical analysis was performed by one-way ANOVA with Tukey’s post hoc test. Body weight data were analysed by two-way repeated measures analysis of variance. * Represents comparison with the control group, # represents comparison with the model group. *P < 0.05, **P < 0.01, ***P < 0.001, and ****P < 0.0001

To evaluate the role of *Nrf2* in CUMS-induced depression, mice were administered the *Nrf2* activator oltipraz. As anticipated, oltipraz administration resulted in a significant increase in body weight (Fig. 4A) and sucrose preference (Fig. 4B). Moreover, oltipraz administration led to an increased total distance moved (Fig. 4C), number of entries into the central area (Fig. 4D), and central area duration (Fig. 4E) in the open field test, along with a reduced immobility time in the TST (Fig. 4F) and FST (Fig. 4G). Additionally, oltipraz treatment significantly reduced iron levels in mice, as demonstrated by decreased levels of serum Fe (Fig. 4H), hippocampal total iron (Fig. 4I) and ferrous iron (Fig. 4J). Given that oltipraz is an effective *Nrf2* activator, we focused on analysing the protein expression of *Nrf2*, which was activated by oltipraz (Fig. 4K and L, Additional file 1: Fig. S3A). Additionally, levels of hippocampal TfR and DMT1, which were upregulated in response to CUMS, were reversed by oltipraz treatment (Fig. 4L and M). To validate the role of *Nrf2* in hippocampal neurosynaptic plasticity, we further detected the expression of an important subunit of AMPAR (a-amino-3-hydroxy-5-methyl-4-isoxazol-propionic acid receptor) involved in synaptic plasticity glutamate receptor 1 (GluR1), PSD95 and SYN by immunostaining. Similarly, oltipraz improved synaptic plasticity by elevating GluR1 (Fig. 4N and O), BDNF (Fig. 4P), PSD95 (Fig. 4Q), and SYN (Fig. 4R), which were reduced by CUMS, illustrating that *Nrf2* activation was involved in the regulation of depression by enhancing neurosynaptic plasticity in the hippocampus. Furthermore, our research indicated that Iba1 staining in the hippocampus of CUMS mice was increased, with activated microglial morphology characterized by enlarged rounded cell bodies and retracted branches, indicative of the active phagocytic state. However, Oltipraz treatment inhibited the activation of microglia (Additional file 1: Fig. S3B). Collectively, these findings suggest that *Nrf2* improves iron deposition by inhibiting TfR and DMT1 and enhances synaptic plasticity by increasing BDNF expression, thereby alleviating depressive-like behaviours.

### *Nrf2*^−/−^ mice showed impaired bilateral-brain functional connectivity

**Fig. 5:**
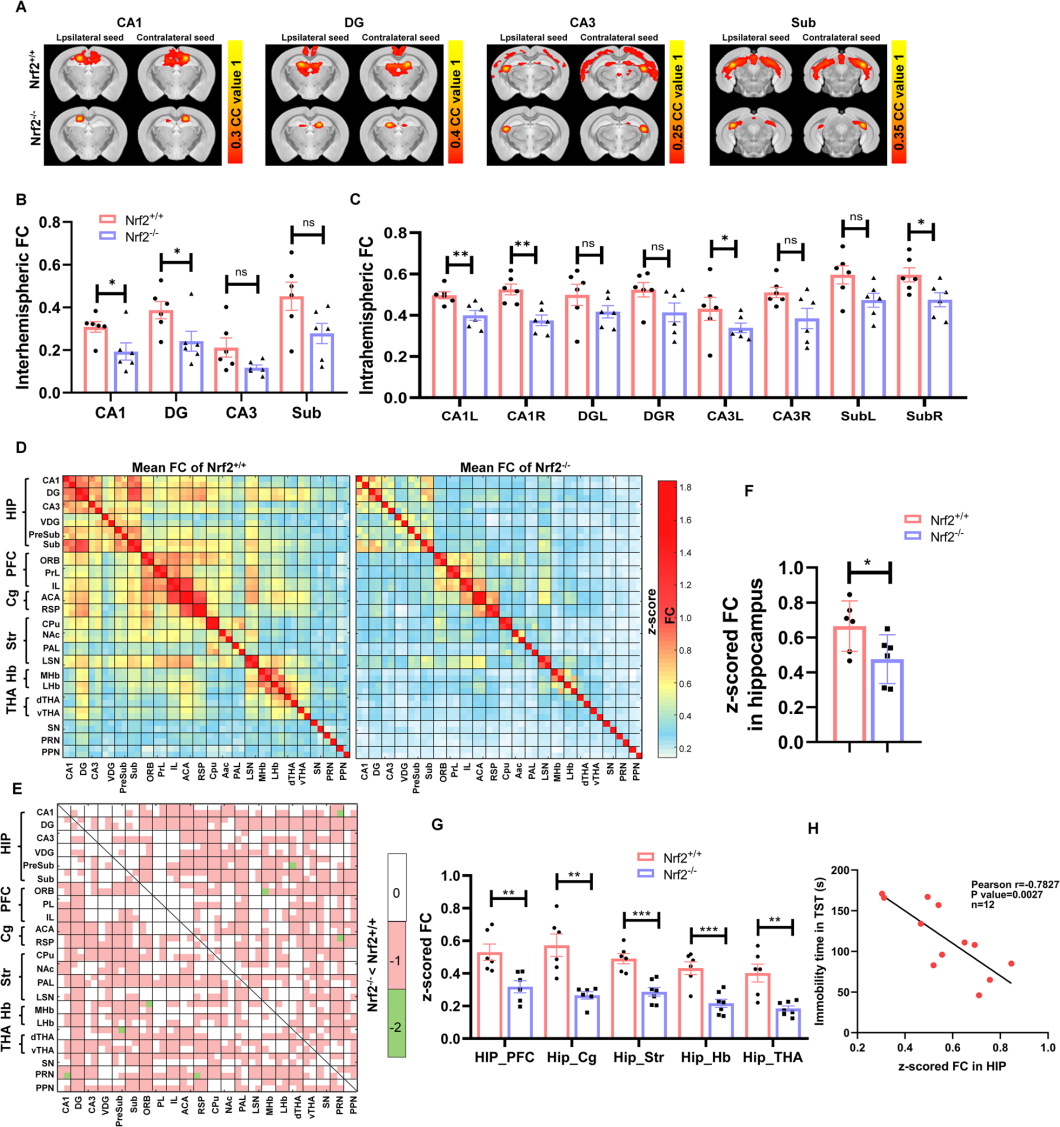
*Nrf2*^*−/−*^ mice showed impaired bilateral-brain functional connectivity. **A** Seed-based analysis represented by functional connectivity maps for *Nrf2*^+/+^ and *Nrf2*^−/−^ mice. The strength of connectivity for the seed region, indicated above each image, is mapped by a colour scale representing the correlation coefficient value (CC) value (abbreviations: CA3 = field of CA3 in hippocampus). **B** and** C** Average interhemispheric and intrahemispheric functional connectivity for ipsilateral and contralateral seeds in *Nrf2*^+/+^ and *Nrf2*^−/−^ mice. **D** Functional connectivity matrices of *Nrf2*^+*/*+^ mice (left) and *Nrf2*^−/−^ mice (right) (postnatal week 12), in which functional correlation (z score) between pairs of regions is represented by a colour scale (abbreviations: ORB = orbital, PL = prelimbic, IL = infralimbic, ACA = anterior cingulate area, RSP = retrosplenial area, Cpu = caudate putamen, NAc = nucleus accumbens, LS*N* = lateral septal nucleus, MHb = medial habenula, LHb = lateral habenula, dTHA = dorsal nucleus of thalamus, vTHA = ventral medial nucleus of the thalamus, S*N* = substantia nigra, PR*N* = pontine reticular nucleus, PP*N* = pedunculopontine nucleus). **E** The functional connectivity matrix of Nrf2^+/+^ and Nrf2^−/−^ mice was subjected to a two-sample T test to obtain a statistically significant matrix, where 0 represents *P* > 0.05, 1 represents *P* < 0.05, and 2 represents *P* < 0.01. **F** Based on the matrix in **D** and **E**, the average functional connectivity strength of the hippocampus with all other regions was calculated for *Nrf2*^+/+^ and *Nrf2*^−/−^ mice. **G** As shown in **D**, functional connectivity was averaged between the hippocampus and the PFC, Cg, Str, Hb, and THA (*n* = 6/group). **H** Pearson linear correlation tests for immobility time in the TST and z scores in the hippocampus. Bars represent the mean ± SEM; statistical analysis was performed by using an unpaired two-tailed *t* test. *P < 0.05, **P < 0.01

Many neuropsychiatric disorders, especially depression, are characterized by impaired brain connectivity. Given that *Nrf2* plays an important regulatory role in depression, we performed fMRI analysis in *Nrf2*^−/−^ mice. Seed-based analysis across brain regions revealed that FC with the contralateral side of the seed region was particularly affected in *Nrf2*^−/−^ mice in the subregions of the hippocampus CA1, DG, CA3, and Sub (Fig. 5A), as well as in the central amygdalar nucleus (CeA), pretectal area (Pre), and pallidum (PAL) (Additional file 1: Fig. S4A). This effect was mainly manifested as a decrease in interhemispheric (Fig. 5B, Additional file 1: Fig. S4B) or intrahemispheric FC (Fig. 5C, Additional file 1: Fig. S3C) values. To further investigate how *Nrf2* insufficiency could affect whole brain networks in vivo, FC in several brain regions using rs-fMRI was analysed, and then the FC values of WT and *Nrf2*^−/−^ mice were analysed by a two-sample T test. *Nrf2*^−/−^ adult mice exhibited decreased FC, especially in the hippocampus, compared to wild-type (WT) mice (Fig. 5D–F). FC networks are characterized by correlations between homotopic regions, which are particularly strong, and midline symmetry [25]. Remarkably, according to Fig. 5E, we observed that the loss of *Nrf2* resulted in weakened FC between the hippocampus and limbic system regions such as the cingulate gyrus, prefrontal cortex, basal ganglia brain regions, striatum, hypothalamus, and habenula (Fig. 5G). In fact, the FC between the hippocampus and the lateral habenula was associated with anhedonic and aversive responses. In addition, attenuated FC in the hippocampus caused by *Nrf2* deletion was strongly associated with the emergence of despair-like behaviours (Fig. 5H). Thus, *Nrf2* deficiency induced weakened FC in the hippocampus, resulting in depressive-like behaviours. In conclusion, our results demonstrated significant defects in FC in *Nrf2*^−/−^ mice, especially in the bilateral connections in the hippocampus.

### Genetic ablation of *Nrf2* aggravated the depression-like phenotypes with or without CUMS exposure in mice by regulating iron metabolism in neurons

**Fig. 6:**
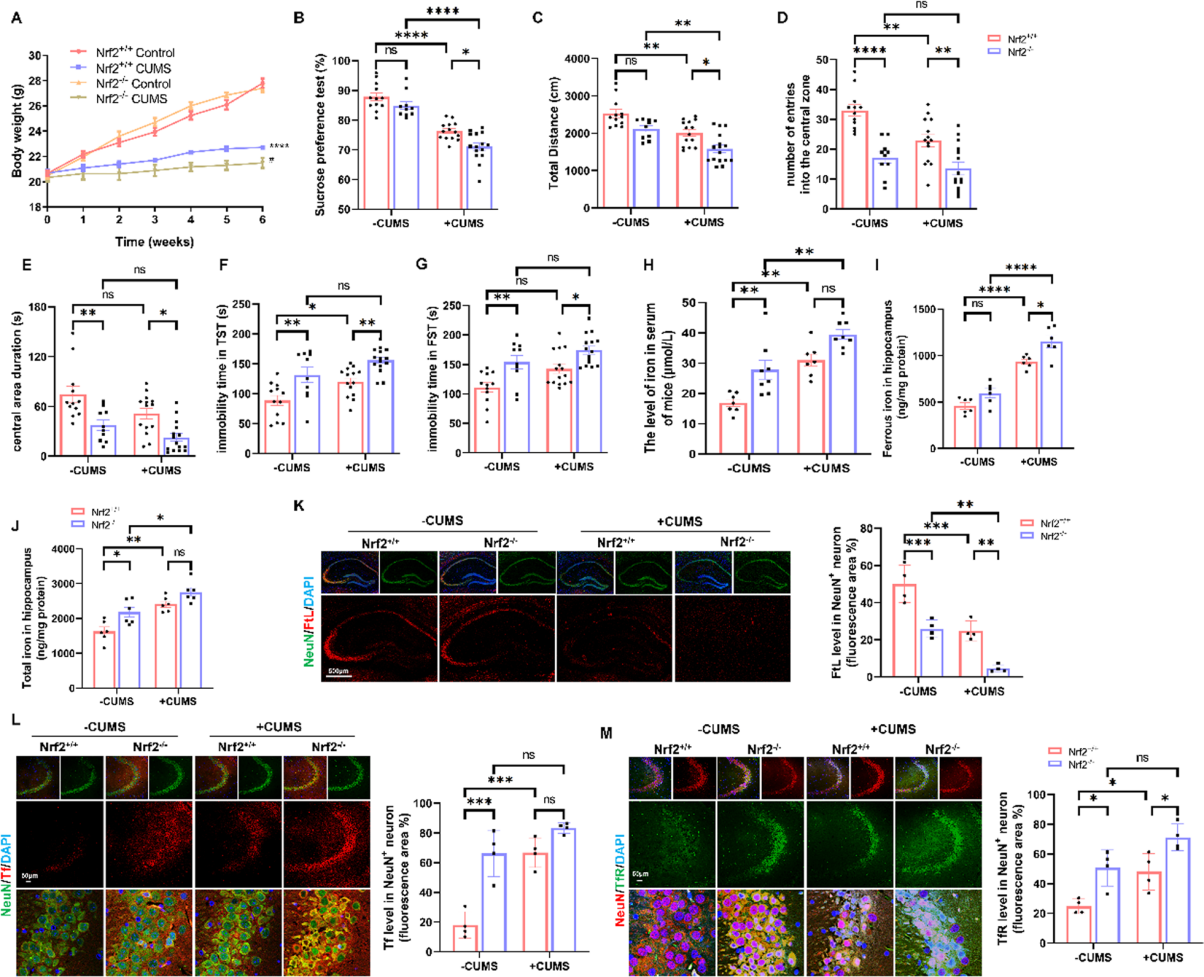
Genetic ablation of *Nrf2* aggravated the depression-like phenotypes with or without CUMS exposure in mice by regulating iron metabolism in neurons. **A–G** Body weight, SPT, OFT, TST and FST in the different indicated groups (control *n* = 12, CUMS *n* = 14, *Nrf2*^−/−^ control *n* = 10, *Nrf2*^−/−^ CUMS *n* = 15 mice). **H** The level of Fe in the serum of mice (control *n* = 7, CUMS *n* = 7, *Nrf2*^−/−^ control *n* = 8, *Nrf2*^−/−^ CUMS *n* = 8 mice).** I** and **J** Ferrous iron and total iron in the hippocampus of the different indicated groups (*n* = 6/group). **K** Immunofluorescence of the hippocampus costained with FtL (red) and the mature neuron marker NeuN (green) (*n* = 4/group; scale bars, 50 μm). **L** Immunofluorescence of the hippocampus costained with Tf (red) and the mature neuron marker NeuN (green) (*n* = 4/group; scale bars, 50 μm). **M** Immunofluorescence of the hippocampus costained with TfR (green) and the mature neuron marker NeuN (red) (*n* = 4/group; scale bars, 50 μm). All images of immunostaining were analysed with ImageJ software. Bars represent the mean ± SEM. Statistical analysis was performed by two-way ANOVA with Bonferroni’s post hoc test. Body weight data were analysed by two-way repeated measures analysis of variance. *Represents comparison with the control group, # represents comparison with the model group. *P < 0.05, **P < 0.01, ***P < 0.001, and ****P < 0.0001

Considering that low expression of *Nrf2* has been associated with depression, *Nrf2*^−/−^ mice with or without CUMS exposure were used to assess behavioural changes and measure body weight and iron metabolism-related proteins. The results demonstrated that WT and *Nrf2*^−/−^ mice exhibited similar body weight gain and sucrose preference (baseline conditions) before CUMS exposure. However, during CUMS, *Nrf2*^−/−^ CUMS mice showed a more unstable body condition, greater weight loss (Fig. 6A), and less sucrose consumption in the sucrose preference test (Fig. 6B) compared to WT CUMS mice, indicating that missing *Nrf2* aggravates the anhedonia induced by CUMS. In addition, the open field test also indicated that *Nrf2*^−/−^ mice were less active and bold than WT mice, as evidenced by the shorter total distance moved (Fig. 6C), fewer entries into the central zone (Fig. 6D) and less time spent in the central arena (Fig. 6E). In the TST and FST (Fig. 6F, G), the immobility time of the *Nrf2*^−/−^ control group was significantly higher than that of the WT control group, suggesting that the knockout of *Nrf2* in mice leads to despair-like behaviour and reduced activity. Furthermore, *Nrf2* knockout aggravated CUMS-induced depression-like behaviours, indicating that *Nrf2* deletion led to depression susceptibility. There was no interaction between CUMS and *Nrf2* ablation. To determine the relationship between *Nrf2* and iron metabolism, iron-related proteins and the level of iron in WT and *Nrf2*^−/−^ mice were assessed. Iron accumulation occurred in *Nrf2*^−/−^ mice with or without CUMS because of the addition of Fe to the serum (Fig. 6H) and higher levels of ferrous iron (Fig. 6I) and total Fe accumulation (Fig. 6J) in the hippocampus. Additionally, we observed a decrease in hippocampal FtL (Fig. 6K) and an increase in Tf (Fig. 6L) and TfR (Fig. 6M) in *Nrf2*^−/−^ mice. Importantly, immunofluorescence colocalization showed that FtL, Tf and TfR were mainly expressed in the cells labelled with NeuN, indicating that *Nrf2* mainly regulates FtL, Tf and TfR in neurons, thereby reducing the occurrence of neuronal iron deposition. Based on these findings, we suggest that *Nrf2* ablation induces depression susceptibility by modulating FtL, Tf and TfR in neurons, leading to aggravated iron overload.

### *Nrf2* deficiency contributed to impaired synaptic plasticity in mice by iron accumulation

**Fig. 7:**
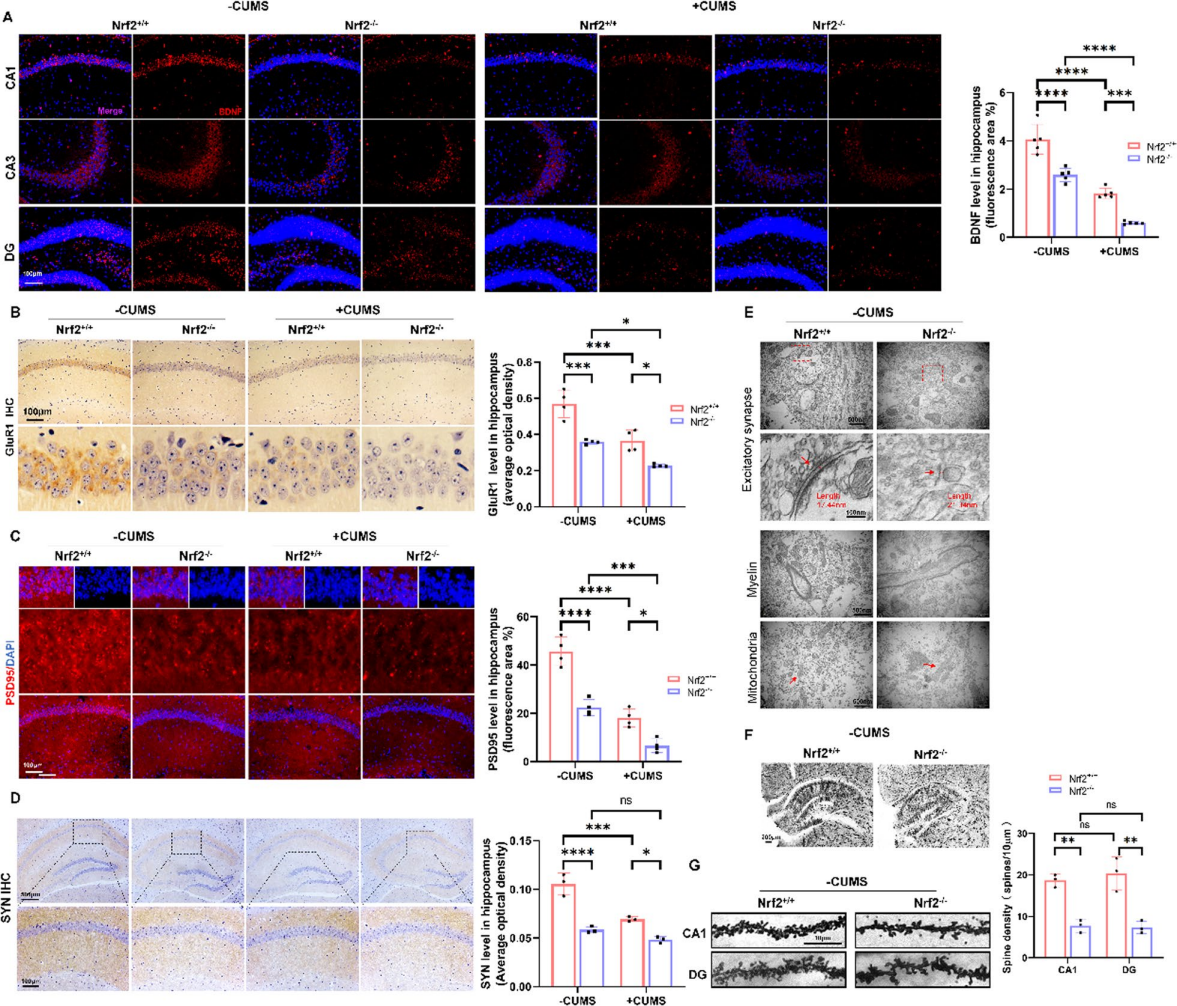
*Nrf2* deficiency contributed to impaired synaptic plasticity in mice by iron accumulation. **A** Immunofluorescence staining of BDNF in the hippocampus. (*n* = 5/group; scale bars, 100 μm). **B** Immunohistochemical staining of GluR1 (sepia) in the hippocampus (*n* = 4/group; scale bars, 100 nm). The AOD of GluR1 staining was measured using ImageJ software (*n* = 4/group). **C** Immunofluorescence staining of PSD95 in the hippocampus (*n* = 4/group; scale bars, 50 μm). **D** Immunohistochemical staining of the microglial marker SYN (sepia) in the hippocampus (*n* = 3/group; scale bars, 100 μm). **E** Electron microscopy image of excitatory/asymmetric spines (arrows point to the synaptic cleft), myelin and mitochondria (*n* = 3/group; scale bars, 500 nm). **F** and** G** Neuroanatomical alterations were analysed in unperfused brains using Golgi staining. The spine density in hippocampal CA1 and DG assessments was analysed by the microscopic image analysis software Imaris (Imaris 8.1, BitPlane) (*n* = 3/group, 15–20 dendrites/mice, per dendritic segments of 10 μm, the scale bars in the dendritic images represent 10 µm). All images of immunostaining were analysed with ImageJ software. Bars represent the mean ± SEM. Statistical analysis was performed by two-way ANOVA with Bonferroni’s post hoc test. *Represents comparison with the control group, # represents comparison with the model group. *P < 0.05, **P < 0.01, ***P < 0.001, and ****P < 0.0001

Further results showed that *Nrf2*^−/−^ mice exhibited synaptic plasticity disorder, as evidenced by significantly reduced levels of BDNF (Fig. 7A), synapse-associated protein GluR1 (Fig. 7B), PSD95 (Fig. 7C) and SYN (Fig. 7D) protein in the hippocampus compared to WT mice. Additionally, the loss of *Nrf2* aggravated CUMS-induced synaptic plasticity disorder. To verify the role of *Nrf2* ablation in hippocampal ultrastructure, changes in synapses, myelin and mitochondria in the hippocampus of WT and *Nrf2*^−/−^ mice were observed using transmission electron microscopy (TEM). Interestingly, compared to WT control mice, *Nrf2*^−/−^ mice exhibited abnormal morphology in synapses and myelin sheaths characterized by reduced synaptic vesicles, an extended synaptic gap, thinner PSD, demyelination and myelin swelling (Fig. 7E). Furthermore, *Nrf2* knockout caused swollen mitochondria in hippocampal neurons, with dissolved and absent cristae, leaving empty intermembrane space (Fig. 7E). Golgi staining could be used to observe neuronal axons, dendrites, and dendritic spines. The results showed that *Nrf2* deletion significantly reduced the number of dendritic branches and the density of dendritic spines in the hippocampal CA1 and DG regions of mice. Moreover, the accumulation and amount of Iba1-labelled microglia (Fig. 7F and G) were significantly higher in the hippocampus of CUMS mice than in controls, and *Nrf2*^−/−^ mice exhibited even larger accumulation than WT mice regardless of CUMS exposure, indicating that *Nrf2* ablation promoted neuroimmune inflammatory cell infiltration and contributed to neuroinflammation in the hippocampus (**Fig. S5A**). Immunofluorescence colocalization of SYN and Iba1 showed that SYN expression was increased in the hippocampal microglia of *Nrf2*^−/−^ mice (Additional file 1: Fig. S5B), suggesting that *Nrf2* deletion promoted the increased phagocytosis of synapses by microglia. In conclusion, *Nrf2* deficiency results in iron overload, contributing to synaptic dysfunction in the hippocampus. Taking the findings above together, we suggest that *Nrf2* plays an important role in brain FC in depression, characterized by regulating the accumulation of iron in neurons and improving synaptic plasticity.

## Discussion

In the present study, we observed a significant inverse relationship between serum iron levels and interhemispheric functional connectivity in the CA1, PreSub and Sub regions of the brain (refer to Fig. 1D). Additionally, our findings indicate an elevation in both ferrous iron and total iron concentrations within the hippocampus of mice exhibiting depressive symptoms. This increase is accompanied by an upregulation of neuronal DMT1 and TfR, as well as the downregulation of FtL. Several studies have documented the entry of iron in the bloodstream into the endothelial cells of the blood brain barrier (BBB). This process occurs either through transferrin receptor-mediated endocytosis of transferrin or independently as nontransferrin-bound iron (NTBI) or as a low molecular weight complex (e.g., citrate, ATP, ascorbate) [26]. Once iron enters the cytoplasmic iron pool of endothelial cells, it is believed to be exported at the abluminal membrane through unknown pathways, potentially involving ferroportin or other transporters. Iron released in the extracellular compartment can be taken up by other cells, such as astrocytes and neurons. Additionally, elevated levels of total iron in the brain may result from inflammation-induced permeability of the BBB. Consequently, inflammatory stimuli in depression, as induced by CUMS, may lead to iron accumulation in neurons [27]. It is well established that neuronal iron supply is tightly regulated by both transferrin-bound iron (TBI) and NTBI [8]. In the pathway of NTBI uptake, ferric iron is reduced to ferrous iron at the cell surface by endogenous ferrireductase. This ferrous iron is then transported into the cytosol via the plasma membrane using a divalent cation transporter such as DMT1 or calcium permeable channels [28, 29]. On the other hand, in the TBI uptake pathway, iron is initially bound to transferrin as ferric iron and subsequently binds to TfR, leading to internalization through endocytosis [30]. Consequently, the elevation of TfR and DMT1, along with the reduction of FtL, inevitably results in the accumulation of labile iron. Our findings suggest that alterations in the labile iron concentration have an impact on the activities of PSD, SYN and NMDAR. The excessive presence of iron is observed to traverse the axons of neurons and result in damage to synapses. Our results demonstrate that the elevation in unstable iron concentrations influences the functioning of PSD, SYN and NMDAR. Impaired plasticity may be responsible for decreased rich-club connectivity [31]. In particular, impaired synaptic plasticity alters the synchrony of both local and distributed neuronal oscillations and could promote brain network dysfunction [32]. Thus, peripheral iron enters the brain through increased TfR and DMT1, leading to iron overload, which damages synapses and then affects brain connectivity.

In the present study, we found a significant involvement of the hippocampus in depression-like behaviour under conditions of stress. By conducting rs-fMRI analysis on mice, we discovered a correlation between elevated serum iron concentration, specifically transferrin-bound iron, and a reduction in interhemispheric FC of the hippocampus. Employing multiple brain regions as regions of interest (ROIs) to assess alterations in brain FC in mice, we determined that the deletion of *Nrf2* leads to decreased functional connectivity in the whole brain, particularly in the FC between the hippocampus and limbic system region. Interestingly, the emergence of despair-like behaviours in *Nrf2*^−/−^ mice was found to be closely correlated with a decrease in FC within the hippocampus. It is widely acknowledged that the hippocampus is highly susceptible to stress and exhibits a degree of plasticity. Previous research conducted on animal models of depression has demonstrated that chronic stress exposure leads to a reduction in hippocampal volume and synaptic plasticity [3, 33]. Previous work utilizing rs-fMRI has demonstrated that genetic susceptibility and chronic stress can lead to changes in the strength of glutamatergic synapses in various regions, including the hippocampus, prefrontal cortex (PFC), and nucleus accumbens (NAc), thereby contributing to the dysfunction of corticomesolimbic reward circuitry [34]. Additionally, a more detailed investigation of the hippocampus revealed that chronic stress induces iron accumulation by inhibiting hippocampal *Nrf2*, resulting in synaptic damage. Recent studies have reported that psychological stress can result in chronic neuroinflammation, leading to iron deposition and impaired iron regulation in the brain [35]. These findings suggest that the hippocampus plays a key role in stress-induced depressive-like behaviours and iron overload, which are regulated by *Nrf2*. Additionally, impairment of hippocampal synaptic plasticity has been identified as a significant neural mechanism underlying depression.

To better understand the relationship between iron overload and synaptic damage in depression, we investigated the effects of the iron chelator DFOM and a high-iron diet in depression induced by CUMS. A high-iron diet contributed to iron deposition along with TfR elevation in the hippocampus, aggravating depression-like behaviours and synaptic damage. Conversely, the iron chelator DFOM reduced iron deposition by decreasing TfR, which attenuated synaptic injury and thus protected against depression. The current study demonstrated that alterations in systemic iron loading contribute to alterations in brain function and structure via a cascade of oxidative stress and inflammation, which contribute to depressive symptoms [34, 35]. Moreover, iron may be transported from the cytosol into vesicles within the synaptic cleft, allowing pre- and post-synaptic membranes to be exposed to free ferrous iron [36]. Evidence indicated that elevated postsynaptic expression of TfR in PAE animals masked the presynaptic effect because it regulated the iron concentration in the synaptic cleft [37]. It was recently described that depletion of iron with a permeable chelator enhances the excitability of hippocampal pyramidal cells, increasing the amount of NMDAR activity [38]. Interestingly, this iron-dependent NMDAR modulation required iron release from lysosomes, which was a DMT1-mediated process [38]. Thus, these findings suggested that TfR-mediated iron overload was intimately associated with synaptic damage in depression.

Deferoxamine (DFO), a hexadentate metal chelator, exhibits a strong affinity for Fe(III), Al(III), and other trivalent metal cations. Recent studies have demonstrated the neuroprotective effects of intranasally administered (IN) DFO in animal models, suggesting its potential as a therapeutic intervention for neurodegenerative diseases. The targeted delivery of drugs to the central nervous system (CNS) through IN administration circumvents the blood‒brain barrier (BBB), which minimizes systemic exposure and reduces associated adverse effects. It has been previously demonstrated that intranasal administration increased the targeting of DFO to the brain compared to intravenous delivery [39]. In our study, DFOM was dissolved in pyrogen-free normal saline solution and administered intranasally three times a week at a dose of 100 mg/kg. It is worth noting that the dosage of DFOM employed in our study was relatively low in comparison to other investigations. Specifically, numerous studies have utilized an intranasal dose of DFO that reached 2.4 mg per mouse or 200 mg/kg in a large number of studies [40, 41]. A study was conducted to measure the concentrations of DFO in the entire body of mice. Following intranasal administration of 24 μL of 2.4 mg of DFO, the concentrations of DFO ranged from 0.2 to 29 μM in the brain, while the concentrations in the blood reached up to 5.2 μM [41]. Given the micromolar blood concentrations and the strong affinity of DFO for free iron, it is evident that intranasal DFO would effectively chelate and decrease the levels of free iron in the blood, leading to systemic effects. One of the limitations of this study was that no concentration gradients were set to find the lowest IN DFO dosing concentration that minimized side effects and systemic effects to achieve the best therapeutic effect.

Cell iron levels are precisely regulated under physiological conditions. It is known that the expression of TfR and ferritin is influenced by the availability of iron, with TfR being downregulated in the presence of elevated iron levels. However, the study has shown that the response of TfR in lactating rat mammary tissue may differ from that in haematopoietic cells in various external iron environments. It has been observed that reticulocytes exhibit a decrease in TfR concentration on the cell surface when submitted to high iron concentrations, whereas mammary tissue displays increased expression of TfR [42]. It is possible that an adaptive mechanism developed in the gland as a response to prolonged feeding of high iron, resulting in the sequestration of iron. Within the central nervous system, glial cells, particularly astrocytes, store up to 75% of ionized iron [9], thereby safeguarding the brain against excessive iron levels [43]. Astrocytes demonstrate the presence of functional TfR in vitro and in vivo, enabling the accumulation of ferric iron [44]. Previous experiments have shown that iron deposition upregulates TFR in astrocytes and triggers reactive astrogliosis [45]. Astrocytes play a defensive role in countering acute iron overload by accumulating iron through both TfR and DMT1, redistributing DMT1 from intracellular locations to the plasmalemma and generating Ca^2+^ signals, which regulate astrocytic reactivity. Normally, astrocytes exhibit greater free iron toxicity and oxidative stress than other brain cells [36]. Thus, astrocytes can activate a transient protective mechanism in response to iron accumulation, enhancing the ability to take up iron to alleviate excessive iron in the synaptic environment [8]. In our study, astrocytes exhibited activation after treatment with CUMS and a high-iron diet. This activation was accompanied by an elevation in the levels of TfR and Tf within the astrocytes, indicating an enhanced capacity for iron absorption in the context of neuronal iron overload (Additional file 1: Fig. S6). Consequently, the reason for the increased TfR in the hippocampus induced by the high-iron diet may be attributed to an upregulation of TFR in astrocytes, which alleviates iron overload within the brain.

Chronic psychological stress is a major risk factor for neuropsychiatric disorders, as it disrupts the stress response and alters neuronal structure. It is well known that chronic psychological stress triggers the activation of the sympathoadrenal‐medullary pathway (SAM) and the hypothalamic–pituitary–adrenal axis (HPA), which regulate the secretion of glucocorticoids (GCs) by the adrenal cortex and the release of neurotransmitters [46, 47]. GCs (cortisol and corticosterone) are the main effector hormones of the HPA axis that regulate the physiological (e.g., metabolic, cardiovascular, and immune) and behavioural (e.g., emotional, cognitive, and motor) responses to stress [48]. The primary GC in rodents is corticosterone (CORT), and its levels exhibit dynamic increases in response to physiological and psychological stressors. The evidence suggests that chronic stress, characterized by a persistent elevation in cortisol serum levels, can have detrimental effects on the intracellular redox balance [49]. It has been suggested that stress hormones have a causal role in impacting oxidative processes induced during the adaptive response [50]. The chronic unpredictable mild stress (CUMS) model serves as a suitable experimental framework for studying chronic stress. The chronic stress induced by CUMS results in elevated levels of GCs and corticosterone, leading to modifications in the redox state [51]. This is supported by research indicating that activation of GR under physiological concentrations of GCs significantly suppresses the transactivation capacity of *Nrf2* [52]. Furthermore, exposure of H4IIE cells to cortisol or cortisone leads to a reduction in Nrf2 activity, which can be reversed by the administration of a GC receptor antagonist or a corticosterone reactivator, specifically the 11-beta-hydroxysteroid dehydrogenase 1 inhibitor, thus counteracting the inhibitory effects of GCs [53]. Thus, CUMS caused unbalanced physiological stress that activated the HPA axis, causing an imbalance between oxidant production and antioxidant capacity, which in turn prevented *Nrf2* activation and reduced *Nrf2* levels.

Here, we conducted a systematic investigation into the role of *Nrf2-*regulated iron accumulation in CUMS-induced depression. In CUMS-induced mice, treatment with the *Nrf2* activator Oltipraz reversed the upregulation of transferrin receptor (TfR) and DMT1, both of which are involved in pro-iron accumulation. In our study, it was observed that hippocampal pyramidal neurons exposed to stress exhibited a decline in *Nrf2* and FtL levels, while TfR and Tf levels were elevated, implying that *Nrf2* plays a direct regulatory role in TfR/Tf and FtL-mediated neuronal iron overload. This conclusion is supported by previous studies indicating that *Nrf2* could regulate the metabolic activities of various iron metabolism-related proteins. Furthermore, the reduction in the storage protein ferritin, which is targeted by *Nrf2*, is associated with an increase in intracellular labile iron*.* Consistent with this process, our results demonstrate that the elimination of *Nrf2* led to the sequestration of labile iron by the reduction of FtL in neurons, as well as the influx of iron into neurons by increased levels of Tf and TfR. This phenomenon plays a significant role in the development of iron overload. These results strongly suggest that the downregulation of *Nrf2*, resulting in the induction of TfR and DMT1, along with the inhibition of ferritin, was a crucial factor in the iron overload induced by CUMS. Therefore, *Nrf2* may be an important factor in maintaining iron homeostasis and a nondepressed state in mice.8

Our findings indicate that the expression of BDNF was hindered and that the levels of synaptic plasticity-related proteins were downregulated, resulting in the manifestation of depression-like behaviour. Our study demonstrates that repeated stress decreased BDNF expression through the facilitation of iron accumulation, consequently leading to impaired hippocampal synaptic plasticity dysfunction. As an important nutrient for neurons, the sustained decreases in BDNF in the mPFC and hippocampus are associated with a decrease in the number and function of synapses, as well as a reduction in synaptic plasticity. This contributes to the impaired coordination between the prefrontal and limbic regions and the occurrence of depression [34, 54]. Therefore, the inhibitory effect of hippocampal iron accumulation on BDNF may be an important pathway in iron-induced synapse damage and weakened hippocampal FC in mice subjected to CUMS. Research has shown that iron accumulation leads to a decrease in hippocampal BDNF levels. However, the utilization of iron chelators has been shown to reverse the reductions in BDNF induced by iron [55]. Consistent with these findings, we demonstrated that DFOM activated *Nrf2* in neurons, resulting in the upregulation of BDNF expression. This activation contributes to the partial prevention of depressive behaviour and restores synaptic function (Fig. 2K, O). In contrast, it was observed that iron overload inhibited BDNF expression in the hippocampus, leading to direct damage to synapses and the manifestation of depression-like behaviours. In addition, numerous studies have demonstrated a correlation between the administration of DFO and *Nrf2* activation, whereas iron overload has been found to hinder *Nrf2* expression [56]. BDNF, one of the *Nrf2* target genes, has been found to exert antidepressant effects in behavioural models of depression [57]. It has been observed that *Nrf2* could bind with the BDNF exon I promotor to activate BDNF transcription [58]. Consequently, inhibition of BDNF expression was observed following the knockout of *Nrf2* (Fig. 7A). Therefore, DFO restored BDNF levels, which may be attributed to the increased expression of *Nrf2* induced by DFOM. Furthermore, the presence of a positive autoregulatory feedback loop in BDNF expression suggests that the decrease in BDNF caused by iron may result in [59] reductions in BDNF transcripts and proteins, thereby aggravating the damage [59]. This mechanism could potentially contribute to iron-associated damage to the structure and function of hippocampal neurons in depression. The collective findings indicate the presence of iron overload in synaptic impairment associated with depression. Additionally, DFOM activated *Nrf2*/BDNF signalling to restore synaptic function, and targeting iron homeostasis with specific medications was a valid therapeutic approach.

Notably, our research found that chronic stimulation-induced iron overload and *Nrf2* depletion resulted in the activation of microglia, exacerbating synapse damage and dendritic spine loss in mice.

The role of microglia, the primary immune effector cells in the hippocampus and a significant source of inflammatory cytokines in the CNS, in mediating the immune response of the CNS in patients with depression has been widely acknowledged and profoundly affects mood and behaviour [60]. Neuroinflammation in depression is characterized by activation of microglia with increased expression of the activation marker translocator protein [61]. Notably, suicide among individuals with major depressive disorder (MDD) is associated with a significant increase in hippocampal microglia [62]. An increasing body of research has shown the interconnectedness between neuronal synaptic damage and plasticity dysregulation with the inflammatory response and the abnormal activation of microglia in depression [63]. Microglia play a significant role as regulators of both structure and function in synaptic plasticity, exerting an influence on neuronal circuitry through various mechanisms, such as synaptic pruning, synaptic stripping, secretion of neurotrophic factors, and modulation of synaptic activity [64–66]. The activation of microglia facilitates the phagocytosis of synaptic material, leading to synaptic loss, which subsequently elicits behaviours resembling depression [67]. Consequently, *Nrf2* depletion results in the activation of microglia, which subsequently leads to a decline in synaptic plasticity in depression. However, the mechanisms through which *Nrf2* regulates microglial phagocytosis necessitate additional investigation.

This study provides insights into the functional implications of *Nrf2* deficiency-mediated iron accumulation on synaptic plasticity and functional connectivity within the brain, particularly in relation to behavioural paradigms that model MDD (as depicted in the Graphical Abstract). We propose that augmented *Nrf2* activity or diminished iron deposition could serve as a biological determinant that adversely interacts with established environmental risk factors associated with MDD. In essence, our findings elucidate a distinct function for *Nrf2* deficiency-induced iron deposition in regulating both brain FC and depression behaviour.

### Supplementary Information

**Additional file 1.**

## Data Availability

The data that support the findings of this study are available from the corresponding author upon reasonable request.

## Abbreviations

*Nrf2*: Nuclear factor-erythroid 2 (NF-E2)-related factor 2
BDNF: Brain-derived neurotrophic factor
CUMS: Chronic unpredicted mild stress
Rs-fMRI: Resting-state functional magnetic resonance imaging
FC: Functional connectivity
ROI: Region of interest
DMT1: Divalent metal transporter 1
Tf: Transferrin
TfR: Transferrin receptor
FTL: Ferritin light chain
DFOM: Deferoxamine mesylate
SPT: Sucrose preference test
OFT: Open-field test
TST: Tail suspension test
FST: Forced swim test
EPI: Echo planar imaging
SYN: Synaptophysin
PSD95: Postsynaptic density-95

## References

[1] Malhi GS, Mann JJ (2018). Depression. Lancet.

[2] Kupfer DJ, Frank E, Phillips ML (2012). Major depressive disorder: new clinical, neurobiological, and treatment perspectives. Lancet.

[3] Duman RS, Aghajanian GK, Sanacora G, Krystal JH (2016). Synaptic plasticity and depression: new insights from stress and rapid-acting antidepressants. Nat Med.

[4] Duman RS, Aghajanian GK (2012). Synaptic dysfunction in depression: potential therapeutic targets. Science.

[5] Yu D, Cheng Z, Ali AI, Wang J, Le K, Chibaatar E, Guo Y (2019). Chronic unexpected mild stress destroys synaptic plasticity of neurons through a glutamate transporter, GLT-1, of astrocytes in the ischemic stroke rat. Neural Plast.

[6] Jiang H, Li X, Chen S, Lu N, Yue Y, Liang J, Zhang Z, Yuan Y (2016). Plasminogen activator inhibitor-1 in depression: results from animal and clinical studies. Sci Rep.

[7] Li G, Zhao M, Cheng X, Zhao T, Feng Z, Zhao Y, Fan M, Zhu L (2020). FG-4592 improves depressive-like behaviors through HIF-1-mediated neurogenesis and synapse plasticity in rats. Neurotherapeutics.

[8] Codazzi F, Pelizzoni I, Zacchetti D, Grohovaz F (2015). Iron entry in neurons and astrocytes: a link with synaptic activity. Front Mol Neurosci.

[9] Gaasch JA, Lockman PR, Geldenhuys WJ, Allen DD, Van der Schyf CJ (2007). Brain iron toxicity: differential responses of astrocytes, neurons, and endothelial cells. Neurochem Res.

[10] Galaris D, Pantopoulos K (2008). Oxidative stress and iron homeostasis: mechanistic and health aspects. Crit Rev Clin Lab Sci.

[11] Richardson A, Heath A, Haszard J, Polak M, Houghton L, Conner T (2015). Higher body iron is associated with greater depression symptoms among young adult men but not women: observational data from the daily life study. Nutrients.

[12] Yao S, Zhong Y, Xu Y, Qin J, Zhang N, Zhu X, Li Y (2017). Quantitative susceptibility mapping reveals an association between brain iron load and depression severity. Front Hum Neurosci.

[13] Huang TL, Lee CT (2007). Low serum albumin and high ferritin levels in chronic hemodialysis patients with major depression. Psychiatry Res.

[14] Gao W, Wang W, Liu G, Zhang J, Yang J, Deng Z (2019). Allicin attenuated chronic social defeat stress induced depressive-like behaviors through suppression of NLRP3 inflammasome. Metab Brain Dis.

[15] Yamamoto M, Kensler TW, Motohashi H (2018). The KEAP1-NRF2 system: a thiol-based sensor-effector apparatus for maintaining redox homeostasis. Physiol Rev.

[16] Zhang JC, Yao W, Dong C, Han M, Shirayama Y, Hashimoto K (2018). Keap1-Nrf2 signaling pathway confers resilience versus susceptibility to inescapable electric stress. Eur Arch Psychiatry Clin Neurosci.

[17] Martin-Hernandez D, Caso JR, Javier MJ, Callado LF, Madrigal J, Garcia-Bueno B, Leza JC (2018). Intracellular inflammatory and antioxidant pathways in postmortem frontal cortex of subjects with major depression: effect of antidepressants. J Neuroinflammation.

[18] Bouvier E, Brouillard F, Molet J, Claverie D, Cabungcal JH, Cresto N, Doligez N, Rivat C, Do KQ, Bernard C (2017). Nrf2-dependent persistent oxidative stress results in stress-induced vulnerability to depression. Mol Psychiatry.

[19] Singh S, Vrishni S, Singh BK, Rahman I, Kakkar P (2010). Nrf2-ARE stress response mechanism: a control point in oxidative stress-mediated dysfunctions and chronic inflammatory diseases. Free Radic Res.

[20] Harada N, Kanayama M, Maruyama A, Yoshida A, Tazumi K, Hosoya T, Mimura J, Toki T, Maher JM, Yamamoto M, Itoh K (2011). Nrf2 regulates ferroportin 1-mediated iron efflux and counteracts lipopolysaccharide-induced ferroportin 1 mRNA suppression in macrophages. Arch Biochem Biophys.

[21] Nairz M, Schleicher U, Schroll A, Sonnweber T, Theurl I, Ludwiczek S, Talasz H, Brandacher G, Moser PL, Muckenthaler MU (2013). Nitric oxide-mediated regulation of ferroportin-1 controls macrophage iron homeostasis and immune function in Salmonella infection. J Exp Med.

[22] Koorts AM, Viljoen M (2007). Ferritin and ferritin isoforms I: structure-function relationships, synthesis, degradation and secretion. Arch Physiol Biochem.

[23] Macara IG, Hoy TG, Harrison PM (1972). The formation of ferritin from apoferritin. Kinetics and mechanism of iron uptake. Biochem J.

[24] Ullmann JF, Watson C, Janke AL, Kurniawan ND, Paxinos G, Reutens DC (2014). An MRI atlas of the mouse basal ganglia. Brain Struct Funct.

[25] Salvador R, Suckling J, Coleman MR, Pickard JD, Menon D, Bullmore E (2005). Neurophysiological architecture of functional magnetic resonance images of human brain. Cereb Cortex.

[26] Ward RJ, Zucca FA, Duyn JH, Crichton RR, Zecca L (2014). The role of iron in brain ageing and neurodegenerative disorders. Lancet Neurol.

[27] Urrutia P, Aguirre P, Esparza A, Tapia V, Mena NP, Arredondo M, González-Billault C, Núñez MT (2013). Inflammation alters the expression of DMT1, FPN1 and hepcidin, and it causes iron accumulation in central nervous system cells. J Neurochem.

[28] Li LB, Chai R, Zhang S, Xu SF, Zhang YH, Li HL, Fan YG, Guo C. Iron exposure and the cellular mechanisms linked to neuron degeneration in adult mice. Cells-Basel 2019; 8. 10.3390/cells8020198 PMC6406573 30813496

[29] Yanatori I, Kishi F (2019). DMT1 and iron transport. Free Radic Biol Med.

[30] Testa U, Pelosi E, Peschle C (1993). The transferrin receptor. Crit Rev Oncog.

[31] Yu M, Engels M, Hillebrand A, van Straaten E, Gouw AA, Teunissen C, van der Flier WM, Scheltens P, Stam CJ (2017). Selective impairment of hippocampus and posterior hub areas in Alzheimer’s disease: an MEG-based multiplex network study. Brain.

[32] Uhlhaas PJ, Singer W (2010). Abnormal neural oscillations and synchrony in schizophrenia. Nat Rev Neurosci.

[33] Price RB, Duman R (2020). Neuroplasticity in cognitive and psychological mechanisms of depression: an integrative model. Mol Psychiatry.

[34] Thompson SM, Kallarackal AJ, Kvarta MD, Van Dyke AM, LeGates TA, Cai X (2015). An excitatory synapse hypothesis of depression. Trends Neurosci.

[35] Wang L, Wang W, Zhao M, Ma L, Li M (2008). Psychological stress induces dysregulation of iron metabolism in rat brain. Neuroscience.

[36] Pelizzoni I, Macco R, Morini MF, Zacchetti D, Grohovaz F, Codazzi F (2011). Iron handling in hippocampal neurons: activity-dependent iron entry and mitochondria-mediated neurotoxicity. Aging Cell.

[37] De La Fuente-Ortega E, Plaza-Briceño W, Vargas-Robert S, Haeger P (2019). Prenatal ethanol exposure misregulates genes involved in iron homeostasis promoting a maladaptation of iron dependent hippocampal synaptic transmission and plasticity. Front Pharmacol.

[38] White RS, Bhattacharya AK, Chen Y, Byrd M, McMullen MF, Siegel SJ, Carlson GC, Kim SF (2016). Lysosomal iron modulates NMDA receptor-mediated excitation via small GTPase, Dexras1. Mol Brain.

[39] Kosyakovsky J, Witthuhn BA, Svitak AL, Frey WN, Hanson LR, Fine JM (2019). Quantifying intranasally administered deferoxamine in rat brain tissue with mass spectrometry. Acs Chem Neurosci.

[40] Guo C, Hao LJ, Yang ZH, Chai R, Zhang S, Gu Y, Gao HL, Zhong ML, Wang T, Li JY, Wang ZY (2016). Deferoxamine-mediated up-regulation of HIF-1α prevents dopaminergic neuronal death via the activation of MAPK family proteins in MPTP-treated mice. Exp Neurol.

[41] Hanson LR, Fine JM, Renner DB, Svitak AL, Burns RB, Nguyen TM, Tuttle NJ, Marti DL, Panter SS, Frey WN (2012). Intranasal delivery of deferoxamine reduces spatial memory loss in APP/PS1 mice. Drug Deliv Transl Res.

[42] Sigman M, Lönnerdal B (1990). Response of rat mammary gland transferrin receptors to maternal dietary iron during pregnancy and lactation. Am J Clin Nutr.

[43] Pelizzoni I, Zacchetti D, Campanella A, Grohovaz F, Codazzi F (2013). Iron uptake in quiescent and inflammation-activated astrocytes: a potentially neuroprotective control of iron burden. Biochim Biophys Acta.

[44] Orre M, Kamphuis W, Osborn LM, Melief J, Kooijman L, Huitinga I, Klooster J, Bossers K, Hol EM (2014). Acute isolation and transcriptome characterization of cortical astrocytes and microglia from young and aged mice. Neurobiol Aging.

[45] Liang S, Lu Y, Li Z, Li S, Chen B, Zhang M, Chen B, Ji M, Gong W, Xia M (2020). Iron aggravates the depressive phenotype of stressed mice by compromising the glymphatic system. Neurosci Bull.

[46] Herman JP, McKlveen JM, Ghosal S, Kopp B, Wulsin A, Makinson R, Scheimann J, Myers B (2016). Regulation of the hypothalamic-pituitary-adrenocortical stress response. Compr Physiol.

[47] Sher LD, Geddie H, Olivier L, Cairns M, Truter N, Beselaar L, Essop MF (2020). Chronic stress and endothelial dysfunction: mechanisms, experimental challenges, and the way ahead. Am J Physiol Heart Circ Physiol.

[48] Smith SM, Vale WW (2006). The role of the hypothalamic-pituitary-adrenal axis in neuroendocrine responses to stress. Dialogues Clin Neurosci.

[49] Aschbacher K, O'Donovan A, Wolkowitz OM, Dhabhar FS, Su Y, Epel E (2013). Good stress, bad stress and oxidative stress: insights from anticipatory cortisol reactivity. Psychoneuroendocrino.

[50] Zafir A, Banu N (2009). Modulation of in vivo oxidative status by exogenous corticosterone and restraint stress in rats. Stress.

[51] López-López AL, Jaime HB, Escobar VM, Padilla MB, Palacios GV, Aguilar F (2016). Chronic unpredictable mild stress generates oxidative stress and systemic inflammation in rats. Physiol Behav.

[52] Kratschmar DV, Calabrese D, Walsh J, Lister A, Birk J, Appenzeller-Herzog C, Moulin P, Goldring CE, Odermatt A (2012). Suppression of the Nrf2-dependent antioxidant response by glucocorticoids and 11β-HSD1-mediated glucocorticoid activation in hepatic cells. PLoS ONE.

[53] Chen HC, Yip T, Lee JK, Juliani J, Sernia C, Hill AF, Lavidis NA, Spiers JG (2020). Restraint stress alters expression of glucocorticoid bioavailability mediators, suppresses Nrf2, and promotes oxidative stress in liver tissue. Antioxidants (Basel).

[54] Failla MD, Juengst SB, Arenth PM, Wagner AK (2016). Preliminary associations between brain-derived neurotrophic factor, memory impairment, functional cognition, and depressive symptoms following severe TBI. Neurorehabil Neural Repair.

[55] Alcalde LA, de Freitas BS, Machado G, de Freitas CP, Dornelles VC, Gus H, Monteiro RT, Kist LW, Bogo MR, Schröder N (2018). Iron chelator deferiprone rescues memory deficits, hippocampal BDNF levels and antioxidant defenses in an experimental model of memory impairment. Biometals.

[56] Chen GH, Song CC, Pantopoulos K, Wei XL, Zheng H, Luo Z (2022). Mitochondrial oxidative stress mediated Fe-induced ferroptosis via the NRF2-ARE pathway. Free Radic Biol Med.

[57] Audet MC, Anisman H (2013). Interplay between pro-inflammatory cytokines and growth factors in depressive illnesses. Front Cell Neurosci.

[58] Cao Q, Zou Q, Zhao X, Zhang Y, Qu Y, Wang N, Murayama S, Qi Q, Hashimoto K, Lin S, Zhang JC (2022). Regulation of BDNF transcription by Nrf2 and MeCP2 ameliorates MPTP-induced neurotoxicity. Cell Death Discov.

[59] Zhang Y, Smolen P, Alberini CM, Baxter DA, Byrne JH (2016). Computational model of a positive BDNF feedback loop in hippocampal neurons following inhibitory avoidance training. Learn Mem.

[60] Ransohoff RM, Brown MA (2012). Innate immunity in the central nervous system. J Clin Invest.

[61] Setiawan E, Wilson AA, Mizrahi R, Rusjan PM, Miler L, Rajkowska G, Suridjan I, Kennedy JL, Rekkas PV, Houle S, Meyer JH (2015). Role of translocator protein density, a marker of neuroinflammation, in the brain during major depressive episodes. Jama Psychiat.

[62] Steiner J, Bielau H, Brisch R, Danos P, Ullrich O, Mawrin C, Bernstein H, Bogerts B (2008). Immunological aspects in the neurobiology of suicide: elevated microglial density in schizophrenia and depression is associated with suicide. J Psychiatr Res.

[63] Mishra A, Kim HJ, Shin AH, Thayer SA (2012). Synapse loss induced by interleukin-1β requires pre- and post-synaptic mechanisms. J Neuroimmune Pharmacol.

[64] Zhou LJ, Peng J, Xu YN, Zeng WJ, Zhang J, Wei X, Mai CL, Lin ZJ, Liu Y, Murugan M (2019). Microglia are indispensable for synaptic plasticity in the spinal dorsal horn and chronic pain. Cell Rep.

[65] Sipe GO, Lowery RL, Tremblay MÈ, Kelly EA, Lamantia CE, Majewska AK (2016). Microglial P2Y12 is necessary for synaptic plasticity in mouse visual cortex. Nat Commun.

[66] Chung WS, Welsh CA, Barres BA, Stevens B (2015). Do glia drive synaptic and cognitive impairment in disease?. Nat Neurosci.

[67] Crider A, Feng T, Pandya CD, Davis T, Nair A, Ahmed AO, Baban B, Turecki G, Pillai A (2018). Complement component 3a receptor deficiency attenuates chronic stress-induced monocyte infiltration and depressive-like behavior. Brain Behav Immun.

