# Supplementary material for: Nrf2 regulates iron-dependent hippocampal synapses and functional connectivity damage in depression
Source: J Neuroinflammation. 2023 Sep 21;20:212. doi: 10.1186/s12974-023-02875-x (PMC10512501; doi:10.1186/s12974-023-02875-x)
Supplement: Supplementary file 1 — Additional file 1. [file 12974_2023_2875_MOESM1_ESM.docx]

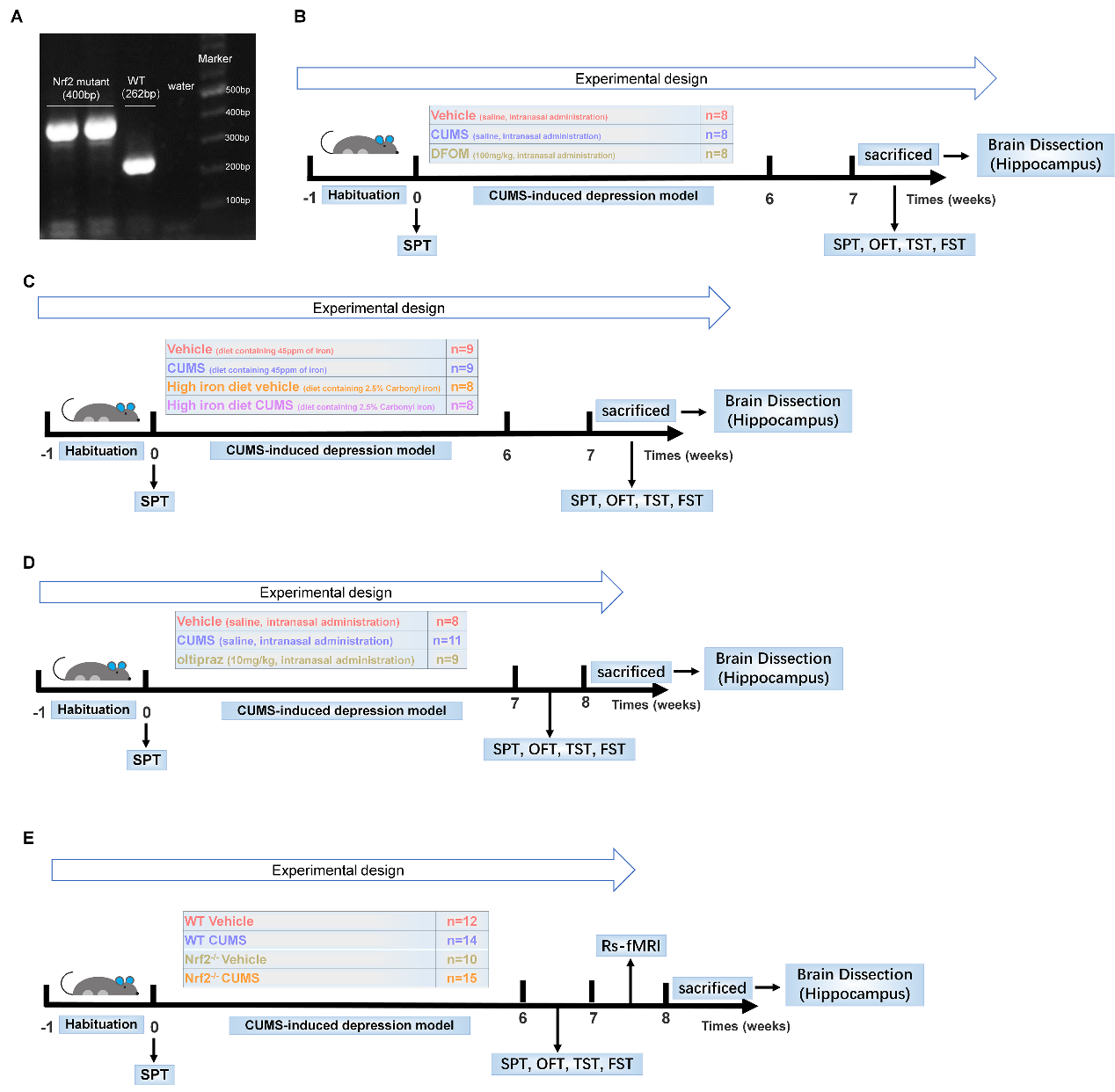


**Fig. S1. Gene identification of *Nrf2*-/- mice and experimental design.**

**(A)** The expected result of *Nrf2*-/-mutant is in the 400bp, while the wild type is in the 262bp.The primer sequences: *Nrf2* Common: GCC TGA GAG CTG TAG GCC C; *Nrf2* Wild Type Reverse: GGA ATG GAA AAT AGC TCC TGC C; *Nrf2* Mutant Reverse: GAC AGT ATC GGC CTC AGG AA. **(B-E)** Schematic showing experimental approach and timeline.


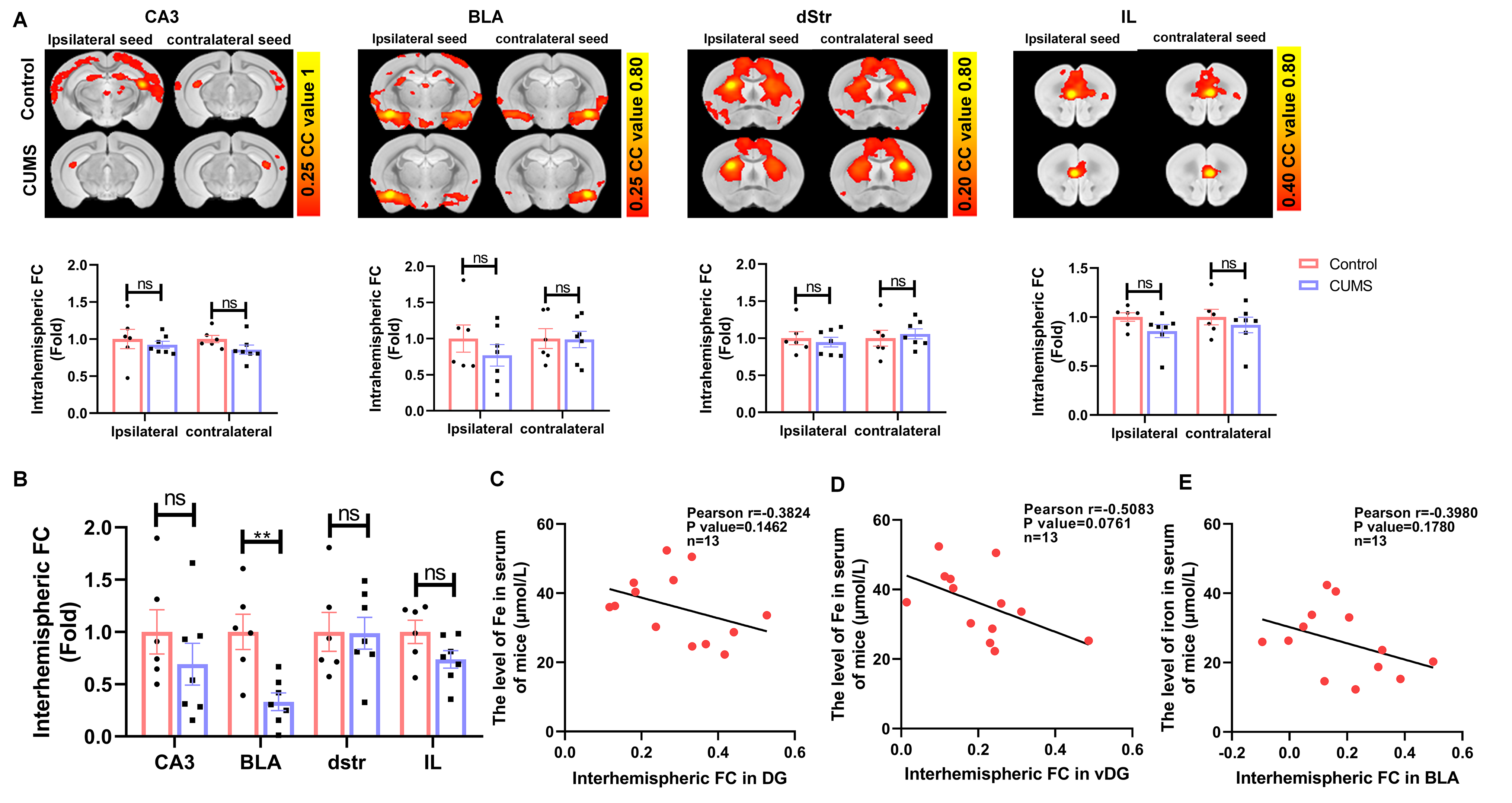


**Fig.S2. Pearson linear correlation tests for iron levels in serum and interhemispheric FC in brain regions, related to Fig.1**

**(A)** Seed-based analysis represented by functional connectivity maps for WT and CUMS mice. The strength of connectivity for the seed region, indicated above each image, is mapped by a colour scale representing the Correlation coefficient value (CC) value (Control n=6 and CUMS n=7; abbreviations: Field of CA3 in hippocampus=CA3, basolateral amygdala=BLA, infralimbic area=IL, striatum=Str). Average interhemispheric functional connectivity for CA3, BLA, IL, Str in Control and CUMS mice. **(B)** Intrahemispheric functional connectivity for lpsilateral and contralateral seed of CA3, BLA, IL, Str in Control and CUMS mice. **(C)**Pearson linear correlation tests for iron levels in serum and interhemispheric FC in DG. **(D)** Pearson linear correlation tests for iron levels in serum and interhemispheric FC in vDG. **(D)** Pearson linear correlation tests for iron levels in serum and interhemispheric FC in BLA.


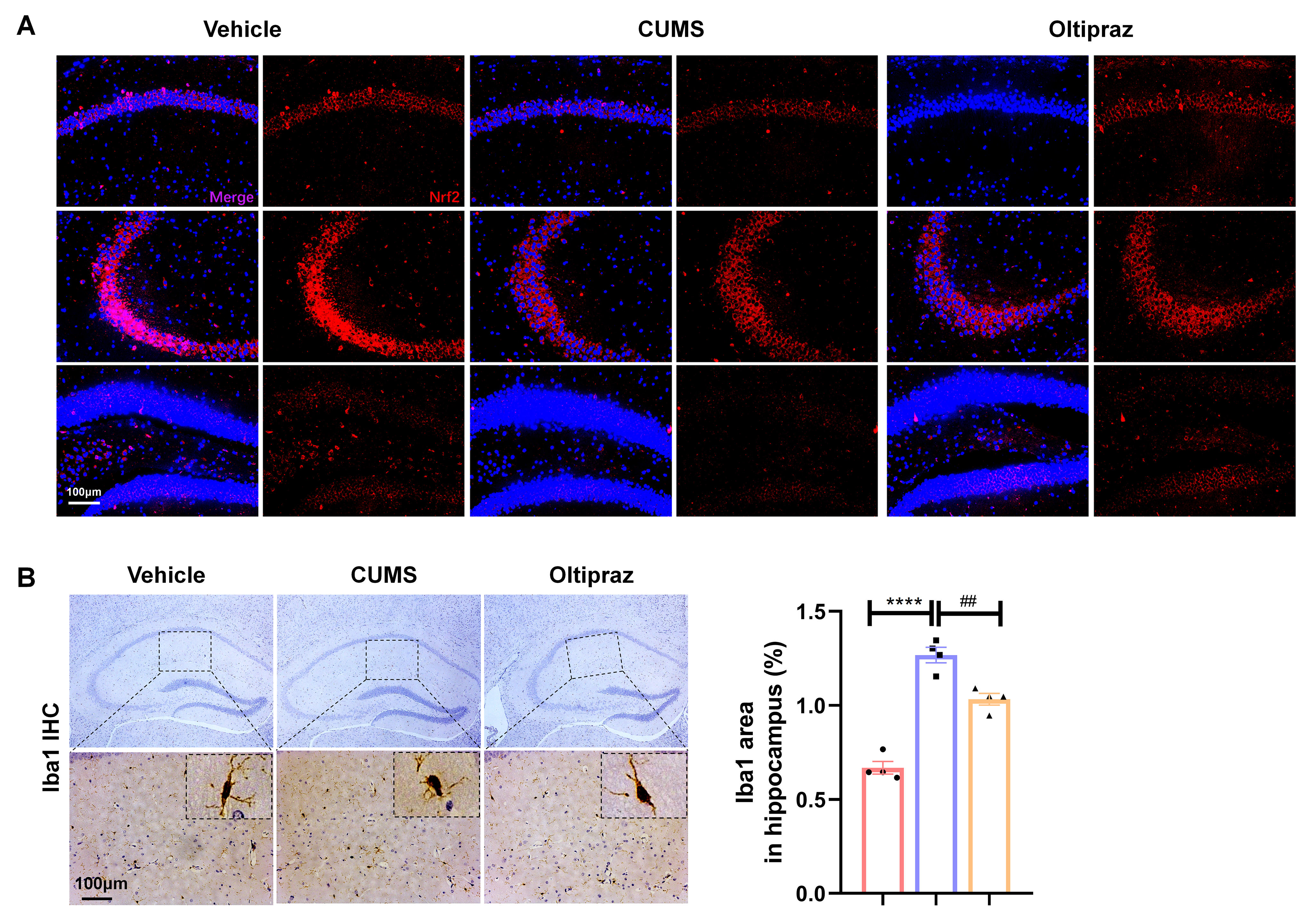


**Fig.S3. oltipraz inhibited microglia activation by increasing *Nrf2* in hippocampus, related to Fig.4**

**(A)** Immunofluorescence staining of *Nrf2* in hippocampus of Vehicle, CUMS and Oltipraz (n=4/group; Scale bars, 100μm).  **(B)** Immunohistochemical staining of microglia markers Iba1 (sepia) in hippocampus (n=4/group; Scale bars, 100μm).


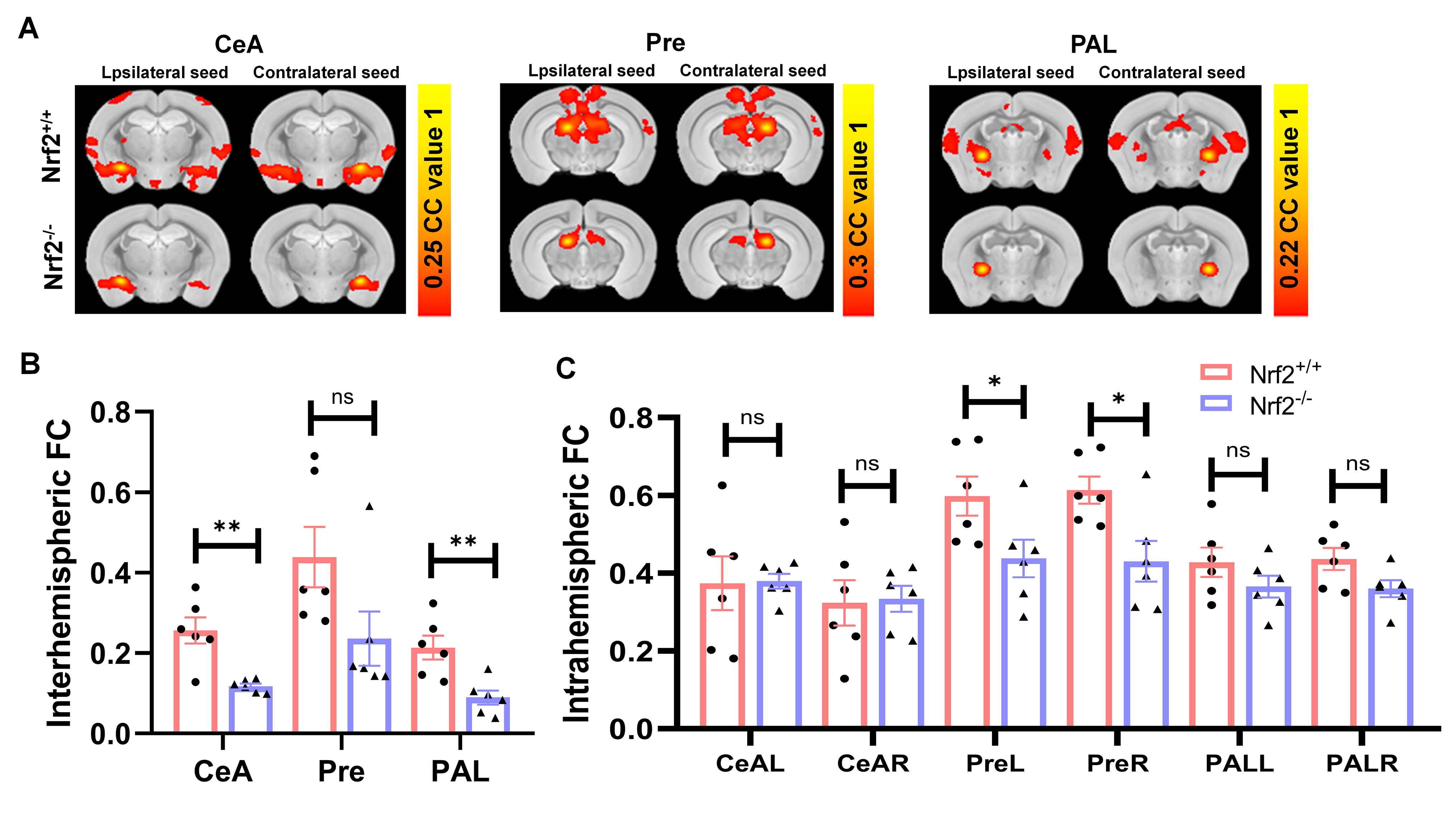


Fig.S4. **Seed-based analysis represented by FC maps for WT and *Nrf2*^-/-^ mice, related to Fig.5**

**(A)**The strength of connectivity for the seed region, indicated above each image, is mapped by a colour scale representing the Correlation coefficient value (CC) value (abbreviations: central amygdalar nucleus=CeA, and pretectal area=Pre, pallidum=PAL). **(B and C)** Average interhemispheric and intrahemispheric FC for CeA, Pre, PAL in Control and *Nrf2*^-/-^ mice (n=6/group). Bars represent mean ± SEM; Statistical analysis was performed by using unpaired two tailed t-test.


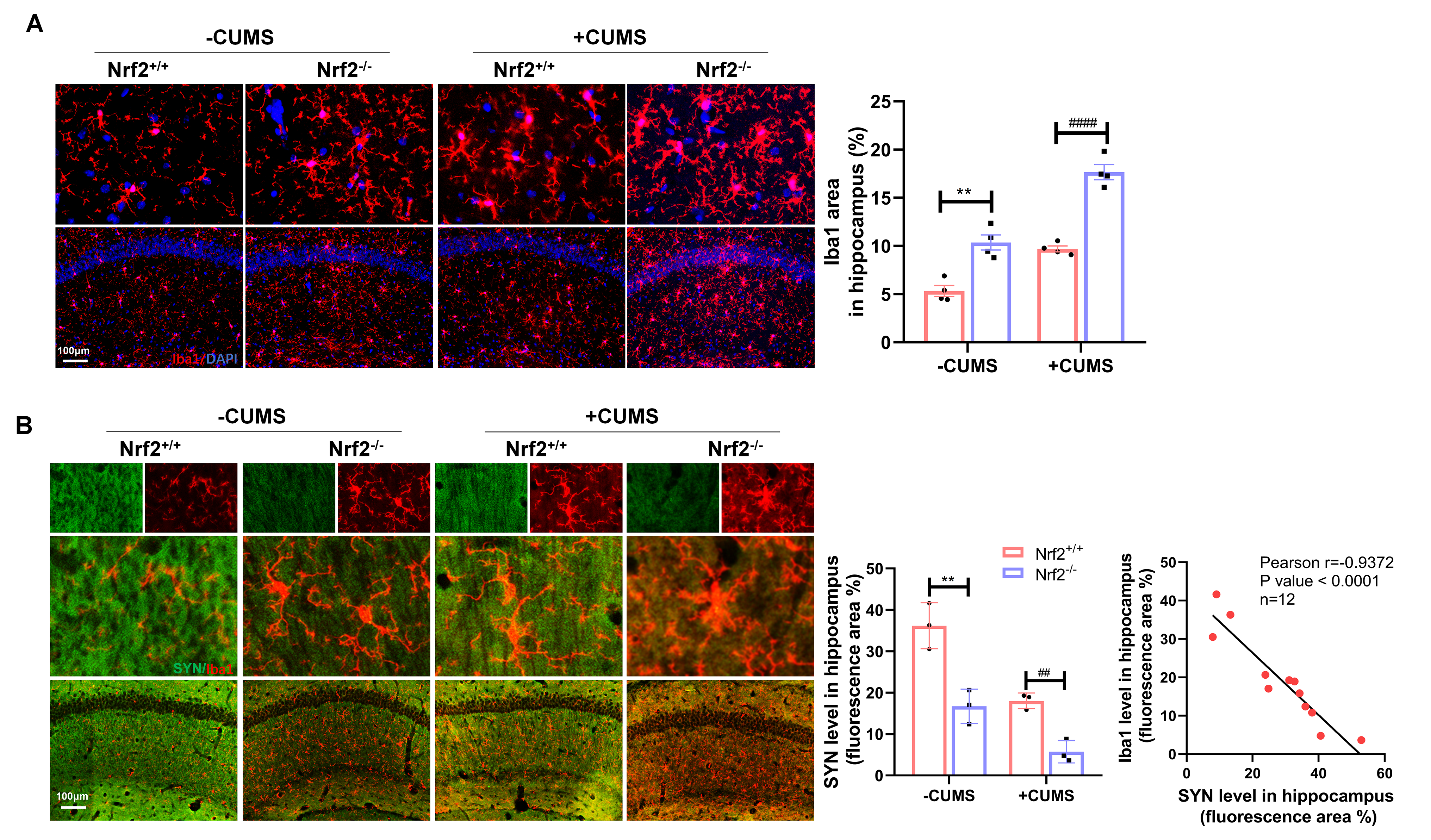


**Fig.S5. *Nrf2* deletion promotes increased phagocytosis of synapses by microglia, related to Fig.7**

**(A)** Immunofluorescence staining of microglia markers Iba1 (sepia) in hippocampus. The Iba1 area in hippocampus were measured using image J software. (n=4/group; Scale bars, 100μm). (**B)** Immunofluorescence of hippocampus co-stained with SYN (green) and the microglia markers Iba1 (red) (n=3/group; Scale bars, 50μm).


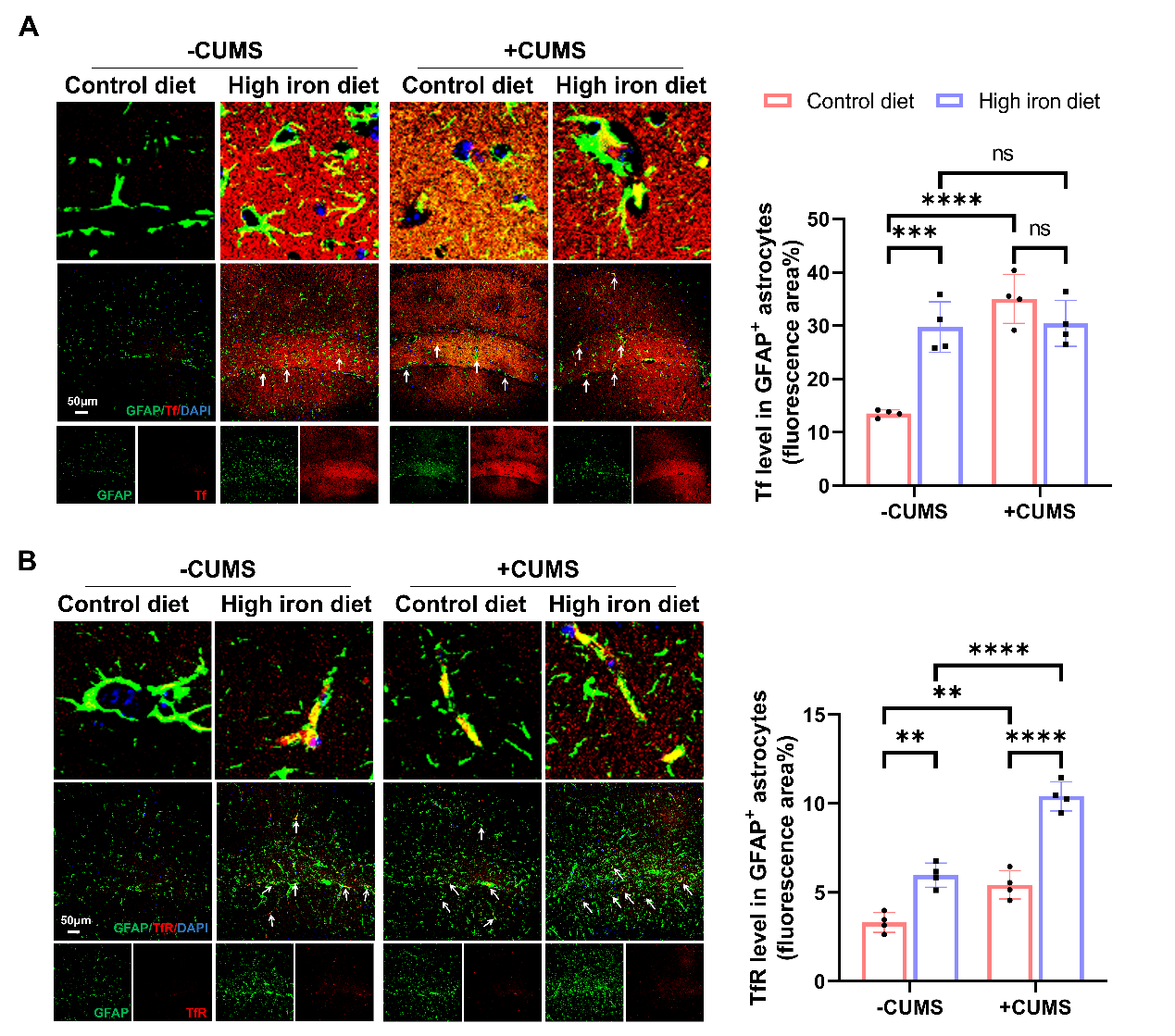


**Fig.S6 A high-iron diet increases TfR and Tf expression in astrocyt.**

**(A)** Immunofluorescence of hippocampus co-stained with Tf (red) and the mature neuron markers GFAP (green) (n=3/group; Scale bars, 50μm). **(B)** Immunofluorescence of hippocampus co-stained with TfR (red) and the mature neuron markers GFAP (green) (n=3/group; Scale bars, 50μm). The fluorescence area of Tf and TfR was measured using image J software (n=3/group; Scale bars, 50μm).
